# Self‐Folding Macromolecular Drug Carrier for Cancer Imaging and Therapy

**DOI:** 10.1002/advs.202304171

**Published:** 2023-11-29

**Authors:** Shan Gao, Yutaka Miura, Akira Sumiyoshi, Satoshi Ohno, Keisuke Ogata, Takahiro Nomoto, Makoto Matsui, Yuto Honda, Minoru Suzuki, Megumi Iiyama, Kensuke Osada, Ichio Aoki, Nobuhiro Nishiyama

**Affiliations:** ^1^ Laboratory for Chemistry and Life Science Tokyo Institute of Technology R1‐11, 4259 Nagatsuta‐cho, Midori‐ku Yokohama Kanagawa 226‐8503 Japan; ^2^ Department of Life Science and Technology School of Life Science and Technology Tokyo Institute of Technology 4259 Nagatsuta‐cho, Midori‐ku Yokohama Kanagawa 226‐8503 Japan; ^3^ Institute for Quantum Medical Science National Institutes for Quantum Science and Technology Anagawa 4‐9‐1, Inage Chiba 263‐8555 Japan; ^4^ Department of Life Sciences Graduate School of Arts and Sciences The University of Tokyo 3‐8‐1 Komaba, Meguro‐ku Tokyo 153‐8902 Japan; ^5^ Division of Particle Radiation Oncology Particle Radiation Oncology Research Center Institute for Integrated Radiation and Nuclear Science Kyoto University 2–1010, Asashiro‐nishi, Kumatori‐cho, Sennan‐gun Osaka 590‐0494 Japan; ^6^ Innovation Center of Nanomedicine (iCONM) Kawasaki Institute of Industrial Promotion 3‐25‐14 Tonomachi Kawasaki Kanagawa 210‐0821 Japan

**Keywords:** contrast agent, delivery system, magnetic resonance imaging, neutron capture therapy

## Abstract

Nano‐sized contrast agents (NCAs) hold potential for highly specific tumor contrast enhancement during magnetic resonance imaging. Given the quantity of contrast agents loaded into a single nano‐carrier and the anticipated relaxation effects, the current molecular design approaches its limits. In this study, a novel molecular mechanism to augment the relaxation of NCAs is introduced and demonstrated. NCA formation is driven by the intramolecular self‐folding of a single polymer chain that possesses systematically arranged hydrophilic and hydrophobic segments in water. Utilizing this self‐folding molecular design, the relaxivity value can be elevated with minimal loading of gadolinium complexes, enabling sharp tumor imaging. Furthermore, the study reveals that this NCA can selectively accumulate into tumor tissues, offering effective anti‐tumor results through gadolinium neutron capture therapy. The efficacy and versatility of this self‐folding molecular design underscore its promise for cancer diagnosis and treatment.

## Graphical abstract

The rare effect of amplifying the relaxation of contrast agents conjugated to a synthetic polymer induced by intramolecular self‐folding is reported. This self‐folding macromolecule loaded with gadolinium complexes can provide specific contrast enhancement of tumors during magnetic resonance imaging and an efficient anti‐tumor effect with neutron capture therapy.

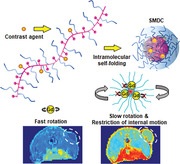

## 1 Introduction

Medical imaging tests are indispensable steps in cancer diagnosis, as they can non‐invasively provide a visual contrast of the internal structures and tumor states of patients. Among diagnostic modalities, magnetic resonance imaging (MRI) has great practicality for providing clues to diseases with high spatial resolution.^[^ ^1^, ^2^ ^]^ Contrast agents (CAs), such as paramagnetic metal complexes, generally serve to manipulate the longitudinal or transverse nuclear relaxation rates of hydrogen nuclei, and macromolecules bound to such metal complexes can facilitate an increase in rotational correlation times, enhancing the molecular relaxivity (slow tumbling effect).^[^ ^3^, ^4^ ^]^ Although the additivity of complexes onto macromolecules and the resulting relaxation rate are not generally established in the current molecular design, the conjugation of CAs and polymers still has the potential to be a versatile platform for increasing image contrast.^[^ ^5^ ^]^ In systemic drug delivery, nano‐sized molecules exhibit long‐term blood circulation with stealth functionality; they selectively accumulate into solid tumors through enhanced permeability and retention effects.^[^ ^6^, ^7^ ^]^ Thus, many molecular architectures, such as liposomes, polymeric micelles, and nanoparticles, have been proposed as effective carriers for delivering CAs to malignant tissues.^[^ ^8^, ^9^, ^10^, ^11^ ^]^ To obtain a clear signal with a low signal‐to‐noise ratio (S/N) in MRI, the concentration balance between the blood and target tissues is critical when using nano‐sized contrast agents (NCAs). This is because excess NCAs in the blood or healthy tissue compartments often confront clear imaging.^[^ ^12^ ^]^ Moreover, prolonged retention of NCAs in the bloodstream increases the risk of Gd^3+^ release and nephrogenic systemic fibrosis.^[^ ^13^ ^]^ Therefore, controlling its blood clearance and accumulation in target tissues is important. The majority of the current NCAs are assemblies of multiple building molecules, leading to dissociation below the critical micelle concentration (CMC) or critical aggregation concentration (CAC) at the last moment of dilution, which occurs after long‐term blood circulation.^[^ ^14^, ^15^ ^]^ However, the detailed fate, influence, and activities of the dissociated components in vivo remain controversial and unclear.^[^ ^16^, ^17^ ^]^

Self‐folding macromolecules are a cornerstone in producing compartmentalized nanomaterials of sub‐10 nm in size with a hydrophobic inner core surrounded by a hydrophilic outer layer.^[^ ^18^ ^]^ Since they are single molecules, CMC/CAC does not exist. The earliest studies were sporadic; however, recent reports have suggested that these nanoscale local environments can induce unique functions, such as restricting the mobility of hydrophilic units around their interface.^[^ ^19^, ^20^ ^]^ Given that such structural properties are applicable for adjusting molecular relaxivity/water exchange and drug delivery to improve in vivo performance, they can lead to further progress in NCA design and applications. Herein, we present the first example of CAs incorporated into a self‐folding macromolecular drug carrier (SMDC) for theranostic applications. Clinically approved gadolinium (Gd)−1,4,7,10‐tetraazacyclododecane‐*N*,*N'*,*N''*,*N'''*‐tetraacetic acid (DOTA) complexes and their derivative were selected for this study. Our results demonstrate that Gd‐DOTA‐incorporated SMDC (SMDC‐Gd) can boost‐up proton longitudinal relaxivity beyond a slow tumbling effect with minimal Gd‐DOTA loading, thereby enabling a lower clinical dose of the contrast agent and reducing the risk of side effects.^[^ ^13^, ^21^, ^22^ ^]^ We also demonstrated the drug delivery and MRI performance of SMDC‐Gd. In this theranostic trial, we present the efficient anti‐tumor effects of Gd neutron capture therapy (Gd‐NCT) using SMDC‐Gd. This highlights the advantages of the molecular design of SMDC over conventional CAs and previous NCA systems.

## 2 Results and Discussion

### 2.1 Design and Characterization of SMDC‐Gd

**Figure 1:**
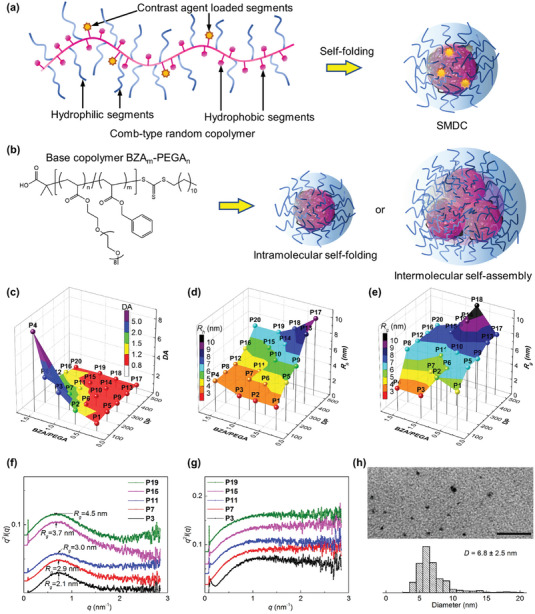
Characterization of the self‐folding macromolecular drug carrier (SMDC). a) Formation of the SMDC from comb‐type random copolymers, in which contrast agents were conjugated on side chains. b) Chemical structure of base copolymers, BZA_m_‐PEGA_n_, and schematic illustrations of the SMDC constructed through intramolecular self‐folding and the polymeric micelle constructed through intermolecular self‐assembly. c) 3D plot showing the degree of aggregation (DA) values of copolymers (**P1**–**P20**) with different degrees of polymerization (DP) and ratios of BZA to PEGA (BZA/PEGA) in water. The color scale (DA) is a secondary representation of the vertical axis of DA. For the calculation of DA values, the molecular weight of copolymers was measured by SEC‐MALS systems in phosphate‐buffered saline (PBS) or chloroform at 40 °C: [copolymer] = 5 mg mL^−1^. 3D plot showing the d) hydrodynamic radius (*R*_h_) and e) radius of gyration (*R*_g_) of copolymers (**P1**–**P20**) with different DP and ratios of BZA/PEGA in water. The color scale (*R*_h_ or *R*_g_) is a secondary representation of the vertical axis of *R*_h_ or *R*_g_. The *R*_h_ was measured by DLS in water at 25 °C, and *R*_g_ was measured by SEC‐MALS in PBS at 40 °C. [copolymer] = 5 mg mL^−1^. f,g) SAXS Kratky plots of copolymers (**P3**, **P7**, **P11**, **P15**, and **P19**) in water or in DMF at 25 °C: [copolymer] = 10 mg mL^−1^. SAXS plots in water indicated the formation of polymeric micelles, and plots in DMF showed the polymer chain condition (no particle). h) TEM image and diameter distribution of the platform copolymer **P7** at 25 °C. TEM sample was prepared from the SMDC aqueous solution (10 mg mL^−1^) on a formvar/carbon‐supported copper grid. The scale bar is 100 nm.

SMDC‐Gd was formed by the intramolecular self‐folding of a comb‐type random copolymer consisting of hydrophobic, hydrophilic, and CA‐loaded segments (**Figure** **1**a). This self‐folding process results in fewer hydrophobic units within a single polymer, establishing a dense core for the SMDC globule. These interactions are intimately tied to the sequences of the base copolymers, leading to varied 3D configurations.^[^ ^19^, ^20^ ^]^ Thus, the key to achieving self‐folding and the creation of SMDC is copolymerization, which modulates the equilibrium between hydrophobic and hydrophilic units. To determine a suitable sequence with controlled molecular weight and verify the feasibility of intramolecular self‐folding, we initiated our study on the synthesis of base copolymers. First, base copolymers were synthesized via reversible addition‐fragmentation chain transfer (RAFT) polymerization using poly (ethylene glycol) methyl ether acrylate (PEGA) and benzyl acrylate (BZA) (Figure 1b). This polymerization gave a series of random copolymers in a controlled manner (Scheme S1, Table S1: **P1**–**P20**, and Figures S1–S5, Supporting Information). For the analysis of the self‐folding behavior in water, Sawamoto and colleagues have investigated it using size‐exclusion chromatography equipped with multi‐angle light scattering (SEC‐MALS),^[^ ^23^ ^]^ and we referred to this method to identify the influence of the total degree of polymerization (DP) and the hydrophobic‐hydrophilic balance (BZA/PEGA) on the degree of aggregation (DA) in water. The DA values indicate the ratio of absolute *M*_w, MALS_ in PBS to absolute *M*_w, MALS_ in chloroform; DA = 1.0, absolute SMDC formation in principle; high DA (DA > 1.0) indicates intermolecular self‐assembly and low DA (DA < 1.0) indicates SMDC formation in water with the potential for reversible micelles formation in organic solvents. The broad scope of the process was demonstrated by the results summarized in Table S2 (Supporting Information). We prepared a 3D distribution map of the DA, DP, and BZA/PEGA ratios, suggesting that a higher DP value with a lower BZA/PEGA ratio led to a DA close to 1.0, but some polymers (**P3**, **P4**, and **P8**) aggregated due to their lack of hydrophilicity (Figure 1c). Hence, a copolymer with a DA below 2.0, was identified as a candidate platform for SMDC in this study because some hydrophilic imaging agents were inserted into the terpolymers in the next step. These tendencies were also investigated using 3D distribution maps of the hydrodynamic radius (*R*_h_) and radius of gyration (*R*_g_) (Figure 1d,e). Contrary to the DA mapping, a higher DP value with a lower BZA/PEGA ratio tended to form large particles in aqueous conditions. Small‐angle X‐ray scattering (SAXS) and transmission electron microscopy (TEM) showed accurate SMDC sizes (Figure 1f–h). Thus, to obtain sub‐10 nm SMDCs in water, we concluded that the appropriate hydrophobic/hydrophilic ratios and DP values were in the range of 4/6–5/5 and 200–400 repeating units, respectively.

**Figure 2:**
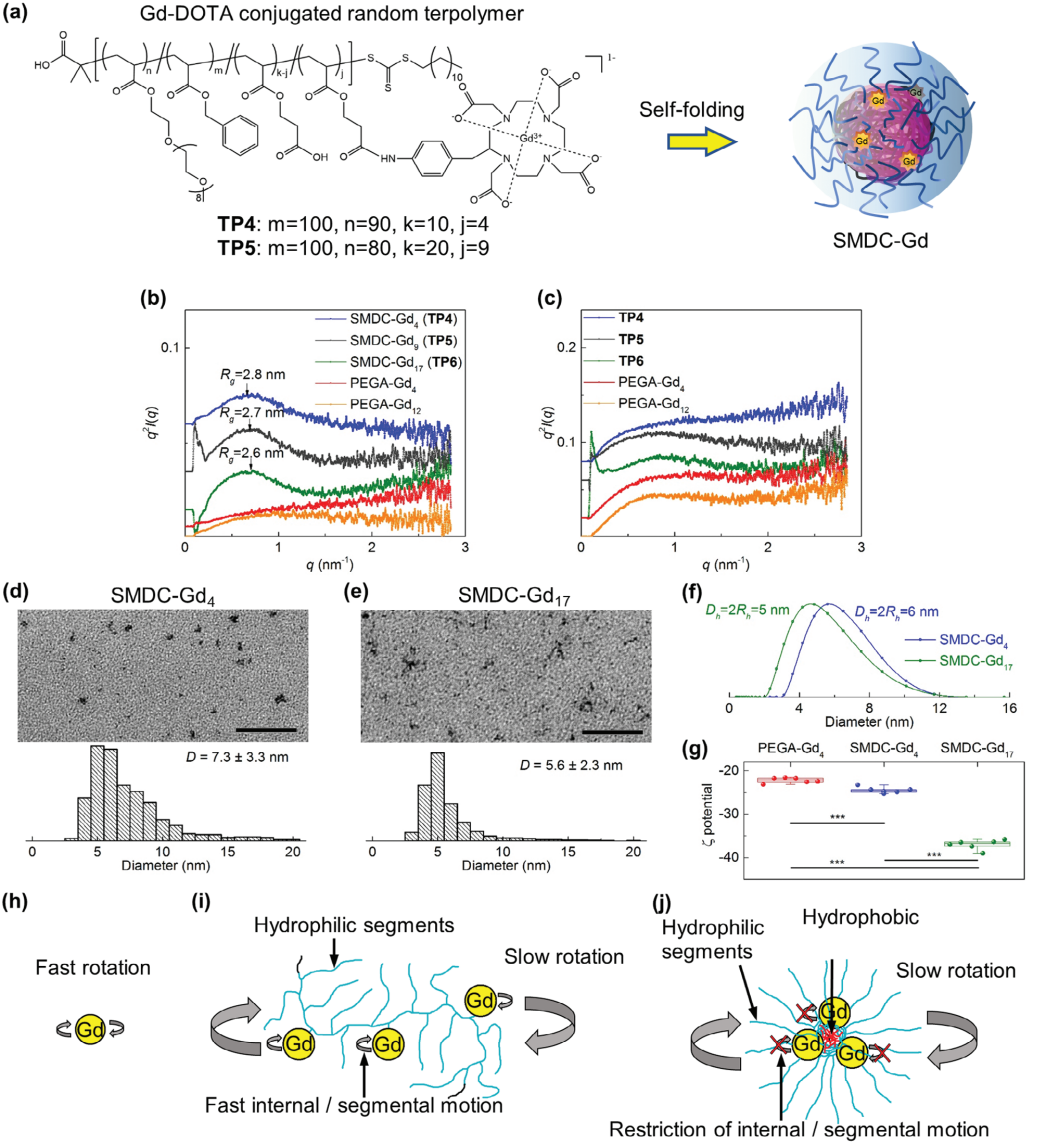
Characterizations of SMDC‐Gds. a) Chemical structure of Gd‐conjugated random terpolymers (**TP4**‐**TP6**), and schematic illustration of the SMDC‐Gd formation. b,c) SAXS Kratky plots of Gd‐loaded copolymers (**TP4**‐**TP6**, and PEGA‐Gds) in water or DMF at 25 °C: [copolymer] = 10 mg mL^−1^. SAXS plots in water showed the formation of SMDC‐Gds by **TP4**‐**TP6**. Plots in DMF demonstrated the reverse micelle formation for **TP5**‐**TP6** and polymer chain conditions (no particle) for **TP4** and PEGA‐Gds. d,e) TEM images and diameter distributions of SMDC‐Gd_4_ and SMDC‐Gd_17_ at 25 °C. TEM samples were prepared from the SMDC‐Gd aqueous solution from the aqueous solutions (10 mg mL^−1^) on a formvar/carbon‐supported copper grid. Scale bars, 100 nm. f) Size distributions of SMDC‐Gds determined by DLS at 25 °C: [copolymer] = 10 mg mL^−1^. g) ζ potentials of SMDC‐Gd_4_, SMDC‐Gd_17_, and PEGA‐Gd_4_ measured by Zetasizer at 25 °C: [copolymer] = 10 mg mL^−1^. Data are shown as box plot, *n* = 6, ^⁎⁎⁎^ *p* < 0.001. h–j) Strategies for increasing the relaxivity. Small molecular Gd complexes performed fast rotation and thus had relatively low relaxivity. Polymer conjugated Gd complexes exhibited slow rotation, but the relaxivity gain was limited by fast internal or segmental motion. SMDC conjugated Gd complexes produced a crowded complex environment, which enabled slow rotation while restricting internal or segmental motion, thus achieved higher relaxivities.

Based on the results above, **P7** was chosen as the platform polymer. All parameters, including the DP value (DP = 200), the hydrophobic/hydrophilic balance (BZA/PEGA = 5/5), and size in water (≈6–8 nm in diameter), pointed toward the favorable formation of SMDCs, even after introducing a small amount of Gd/DOTA complexes to the side chain. To validate this, a terpolymer was synthesized via RAFT polymerization using PEGA, BZA, and 2‐carboxyethyl acrylate (CEA) (Scheme S4, Supporting Information). The reactivity and randomization of each monomer in radical copolymerization were separately confirmed by a polymerization reaction with BZA_m_‐CEA_k_ and PEGA_n_‐CEA_k_ (Schemes S2 and S3, Figures S6–S9, Supporting Information). Random terpolymers such as BZA_100_‐PEGA_90_‐CEA_10_ (**TP1**), BZA_100_‐PEGA_80_‐CEA_20_ (**TP2**), and BZA_100_‐PEGA_70_‐CEA_30_ (**TP3**) were prepared in a controlled living manner (Figures S10–S12, Supporting Information). The moiety of *S*‐2‐(4‐aminobenzyl)‐DOTA was inserted into **TP1**‐**TP3** by a condensation reaction, and Gd chelates were formed on the side chain of the DOTA‐conjugated terpolymers (**Figure** **2**a: **TP4**‐**TP6**, Scheme S5, Figure S13, Supporting Information): **TP4** was equipped with four molecules of Gd‐DOTA, **TP5** was equipped with nine molecules of Gd‐DOTA, and **TP6** was equipped with 17 molecules of Gd‐DOTA. Gd‐DOTA‐conjugated copolymers without hydrophobic components were prepared as control samples (PEGA‐Gd_4_ and PEGA‐Gd_12_; Scheme S6 and Figure S14, Supporting Information). The obtained **TP4**‐**TP6** maintained the ability to self‐fold to form SMDC‐Gd_4_, SMDC‐Gd_9_, and SMDC‐Gd_17_ based on SAXS, SEC‐MALS, DLS, and TEM. For **TP4**–**TP6**, SAXS plots showed the existence of particles in the aqueous solution (Figure 2b), and DA values from SEC‐MALS were equal to 0.91, 0.88, and 0.53 (Table S3, Supporting Information), suggesting the successful formation of SMDC‐Gds; in contrast, PEGA‐Gds and organic solvent systems did not show any particle formation (Figure 2c). For **TP4** and **TP6**, the TEM images, quantified data, and SAXS showed that both obtained SMDC‐Gds were sub‐10 nm in diameter, ranging from ≈5.0–7.0 nm with narrow distributions, which was consistent with the DLS analysis (Figure 2d–f).

Although numerous polymeric micelles have been developed for drug delivery applications and studies have shown that a smaller size (e.g., 30–100 nm in diameter with CMC/CAC) provided good accumulation, penetration, and extravasation against malignant tumors,^[^ ^24^ ^]^ the construction of sub‐10 nm‐sized drug carriers without CMC/CAC has not been achieved through polymer engineering. This size range is crucial because urinary excretion is one of the main systems for nanoparticle clearance after systemic administration; glomerular capillaries and/or renal filters also have diameter‐related thresholds. Liver accumulation can also be controlled to 8–9 nm.^[^ ^25^, ^26^ ^]^ The size of our SMDC‐Gds was designed to be slightly larger than critical renal values. The obtained SMDC‐Gds also had negative zeta potentials (Figure 2g), driven by the carboxylic acid residues on the polymer side chains. This electrically negative surface charge is also an important feature for drug carriers to prevent nonspecific interactions with plasma proteins and avoid quick clearance by the mononuclear phagocyte system when SMDC‐Gds are systemically administrated into the bloodstream. Additionally, we assessed the stability and potential Gd leakage of SMDC‐Gds. After ten‐day incubation under physiological conditions, specifically in HEPES buffer (1 m, pH 7.4 at 37 °C) with NaCl (150 mm) and bovine serum albumin (BSA) (10 mg mL^−1^), there was negligible release of Gd^3+^ ions from SMDC (Figure S15, Supporting Information). This suggests that SMDC‐Gds have suitable resilience as systemically injectable NCAs.

We then investigated the relationship between relaxivity and SMDC formation. In general, the binding of CAs, such as Gd‐chelate complexes, to the surface of NCAs can maintain positive signal enhancement in *T*_1_‐weighted imaging, although the increase in longitudinal relaxivity (*r*_1_) is limited. The binding of Gd‐chelate complexes to the interior of NCAs induces relaxivity; however, the capability to obtain positive signal enhancement in MR images is sacrificed due to hydrophobicity and increased transverse relaxivity (*r*_2_). The measured relaxation parameters, including *r*_1_ and *r*_2_, along with the relaxometric ratio, *r*_1_/*r*_2_, at the two distinct magnetic fields are detailed in **Table** **1** and **2**. Compared to clinically approved Gd‐DOTA, SMDC‐Gds and PEGA‐Gds showed higher *r*_1_ and *r*_2_ values at both 0.47 and 1.5 T due to slowed down rotation as a result of conjugation with the polymer and CAs, leading to an increased molecular weight.^[^ ^27^, ^28^ ^]^ We also found that SMDC‐Gds exhibited higher *r*_1_ and *r*_2_ values than PEGA‐Gds. Tethering of the Gd‐DOTA complexes onto the polymer enabled an increase in relaxivity (slow tumbling effect).^[^ ^29^, ^30^ ^]^ However, the effect of increasing the amount of Gd is negligible owing to fast segmental motion or internal motion.^[^ ^27^, ^28^ ^]^ A notable finding is that the self‐folding process of SMDC‐Gds produced a crowded complex environment (i.e., a slow rotation environment), further facilitating the increase in relaxivities at both 0.47 and 1.5 T because of possible restrictions of internal/segmental motion around the interface of SMDC‐Gds (Figure 2h–j). Little effect was attributed to the increasing number of Gd‐DOTA molecules within the same formulation, suggesting that SMDC‐Gd could potentially induce high relaxivity with a small Gd payload. To utilize SMDC‐Gd as a systemically injectable NCAs, its antifouling property and maintenance of positive effects (high *r*_1_/*r*_2_) in the bloodstream are fundamental. Thus, the effects of bovine serum albumin (10 mg mL^−1^) on *r*_1_, *r*_2_, and *r*_1_/*r*_2_ were assessed. Imperceptible effects were observed for SMDC‐Gds and PEGA‐Gd_4_, illustrating almost no protein interaction with SMDC‐Gds and PEGA‐Gd due to PEG shielding. We also examined the impact of the magnetic field on relaxation parameters. Typically, the *r*_1_ value of Gd‐chelates peaks at ≈0.5 T and then exhibits a decline at higher field strengths.^[^ ^31^ ^]^ However, the *r*_1_ values for our SMDC‐Gds displayed minor decreases at 1.5 T (Table 2). Furthermore, *r*_2_ generally rises with increased magnetic fields, a trend that may be more pronounced in nanoparticle applications. Yet, the *r*_2_ of our SMDC‐Gds displayed only marginal variations between 0.47 and 1.5 T (Table 1 and 2), hinting at potential challenges in *r*_2_ augmentation due to the self‐folding process. While our measurements were exclusively conducted at 0.45 and 1.5 T, these magnetic field strengths are frequently utilized in clinical environments. As such, the aforementioned findings underscore the potential utility of SMDC‐Gds as a reliable macromolecule contrast agent.

**Table 1:** Relaxivities and relaxometric ratios of Gd‐conjugated contrast agents in water and bovine serum albumin (BSA) aqueous solutions at 0.47 T magnetic field.

	*r*_1_ ^a)^ [s^−1^mm ^−1^]	*r*_2_ ^b)^ [s^−1^mm ^−1^]	*r*_1_/*r*_2_ ^c)^	*r*_1_ with BSA^d)^ [s^−1^mm ^−1^]	*r*_2_ with BSA^e)^ [s^−1^mm ^−1^]	*r*_1_/*r*_2_ with BSA^f)^
Gd‐DOTA^[^ ^35^ ^]^	3.4	4.1	0.83	–	–	–
PEGA‐Gd_4_	19.6 ± 0.1	23.6 ± 0.1	0.83	21.4 ± 0.1	25.9 ± 0.1	0.83
PEGA‐Gd_12_	20.4 ± 0.1	23.0 ± 0.1	0.89	–	–	–
SMDC‐Gd_4_ (**TP4**)	25.9 ± 0.1	31.1 ± 0.1	0.83	26.6 ± 0.1	32.1 ± 0.1	0.83
SMDC‐Gd_9_ (**TP5**)	22.8 ± 0.1	28.0 ± 0.1	0.81	–	–	–
SMDC‐Gd_17_ (**TP6**)	24.7 ± 0.1	29.6 ± 0.1	0.83	25.5 ± 0.1	30.8 ± 0.1	0.83

^a)^ Longitudinal relaxivity in water at 37 °C;

^b)^ Transverse relaxivity in water at 37 °C;

^c)^ Relaxometric ratio in water at 37 °C;

^d)^ Longitudinal relaxivity in 10 mg mL^−1^ BSA aqueous solution at 37 °C;

^e)^ Transverse relaxivity in 10 mg mL^−1^ BSA aqueous solution at 37 °C;

^f)^ Relaxometric ratio in 10 mg mL^−1^ BSA aqueous solution at 37 °C.

**Table 2:** Relaxivities and Relaxometric Ratios of Gd‐Conjugated Contrast Agents in Water and Bovine Serum Albumin (BSA) Aqueous Solutions at 1.5 T Magnetic Field.

	*r*_1_ ^a)^ [s^−1^mm ^−1^]	*r*_2_ ^b)^ [s^−1^mm ^−1^]	*r*_1_/*r*_2_ ^c)^	*r*_1_ with BSA^d)^ [s^−1^mm ^−1^]	*r*_2_ with BSA^e)^ [s^−1^mm ^−1^]	*r*_1_/*r*_2_ with BSA^f)^
Gd‐DOTA^[^ ^35^ ^]^	2.9	3.2	0.91	–	–	–
PEGA‐Gd_4_	15.1 ± 0.1	20.4 ± 0.1	0.74	15.0 ± 0.1	20.9 ± 0.1	0.72
PEGA‐Gd_12_	14.7 ± 0.1	19.9 ± 0.1	0.74	–	–	–
SMDC‐Gd_4_ (**TP4**)	17.8 ± 0.1	30.3 ± 0.1	0.59	18.3 ± 0.1	31.8 ± 0.1	0.58
SMDC‐Gd_9_ (**TP5**)	17.8 ± 0.1	29.9 ± 0.1	0.60	–	–	–
SMDC‐Gd_17_ (**TP6**)	18.3 ± 0.1	31.5 ± 0.1	0.58	18.3 ± 0.1	32.6 ± 0.1	0.56

^a)^ Longitudinal relaxivity in water at 37 °C;

^b)^ Transverse relaxivity in water at 37 °C;

^c)^ Relaxometric ratio in water at 37 °C;

^d)^ Longitudinal relaxivity in 10 mg mL^−1^ BSA aqueous solution at 37 °C;

^e)^ Transverse relaxivity in 10 mg mL^−1^ BSA aqueous solution at 37 °C;

^f)^ Relaxometric ratio in 10 mg mL^−1^ BSA aqueous solution at 37 °C.

### 2.2 In Vivo Performance of SMDC‐Gd

**Figure 3:**
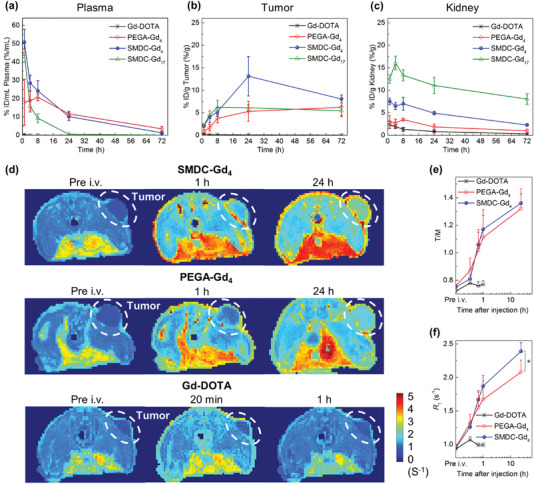
Tumor‐targeted accumulation and contrast enhancement for cancer diagnostics. a–c) Time profiles of Gd concentrations in plasma, tumors, and kidneys in mice after intravenous administration of Gd‐conjugated contrast agents at the dose of 5 mg kg^−1^ on Gd basis. Data are shown as mean ± s.d., *n* = 6. The blood circulation and clearance behaviors were presented by Gd concentrations in plasma. Meanwhile, time‐dependent tumor accumulation of SMDC‐Gds was observed, and SMDC‐Gd_4_ achieved the highest Gd concentration (13.1 ± 4.4% ID/g) in tumors at 24 h after administration. d) Quantitative longitudinal relaxation rate (*R*_1_, 1/*T*_1_, s^−1^) maps of CT26 tumor‐bear mice at 1‐tesla MRI, *n* = 4. Compared to the results of Gd‐DOTA and PEGA‐Gd_4_, the contrast in the tumor (white dashed area) was selectively enhanced by using SMDC‐Gd_4_ at both 1 and 24 h after intravenous administration. e,f) Comparison of tumor‐to‐muscle *R*_1_ ratios (T/M) and *R*_1_ values in tumor areas showed the enhancement of signals in tumor areas (white dashed area in (d) by SMDC‐Gd_4_. Data are shown as mean ± s.d., *n* = 4, ^⁎^ *p* < 0.05.

Research on biodistribution using the SMDC system can clarify the advantages of self‐folding for tumor‐targeting applications and MRI detection. In this study, SMDC‐Gds were tested against xenografted murine colon carcinoma 26 (CT26)‐bearing mice and compared with PEGA‐Gd_4_ and Gd‐DOTA. The profiles of plasma clearance represented a comparable ability of blood circulation and clearance for SMDC‐Gd_4_ and PEGA‐Gd_4_, whereas SMDC‐Gd_17_ showed relatively rapid elimination (**Figure** **3**a). Enhanced tumor accumulation was achieved for SMDC‐Gd_4_, with Gd concentration greater than two‐fold higher, compared to that of SMDC‐Gd_17_ and PEGA‐Gd_4_ 24–48 h after administration (Figure 3b). As expected, Gd‐DOTA showed rapid blood clearance and low accumulation in the tumor model. These observations strongly suggest that self‐folding could provide a PEGylated outer layer around the SMDC, allowing anti‐aggregation and anti‐protein‐binding functions after intravenous injection. For SMDC‐Gd_4_, the accumulation trends in other major organs were comparable to those of typical nanomedicines.^[^ ^32^, ^33^, ^34^ ^]^ Meanwhile, SMDC‐Gd_17_ accumulated to a greater extent in the liver, spleen, and kidneys (Figure 3c; Figures S15–S19, Supporting Information). SMDC‐Gd_4_ and SMDC‐Gd_17_ had a difference of ≈1 nm in diameter but a 1.5‐fold difference in *ζ* potential values. Moreover, SMDC‐Gd_4_ and PEGA‐Gd_4_ exhibit similar *ζ* potentials. Because the physicochemical properties of drug carriers critically affect their fate in the body, molecular design, size control, and surface charge are important features. Generally, neutral and slightly negative surface charges can prevent nonspecific interactions with plasma proteins to avoid rapid clearance by the mononuclear phagocyte system. However, SMDC‐Gd_17_ (*ζ* potential = −37 mV) exhibited an exceedingly negative charge, leading to proactive uptake by hepatic nonparenchymal cells, such as Kupffer cells and macrophages, through scavenger receptor‐mediated endocytosis. Analysis of the area under the curve (AUC) and AUC ratio revealed that SMDC‐Gd_4_ selectively accumulated in tumors over time, and the quick blood clearance of SMDC‐Gds contributed to the increase in tumor/major organ (liver, spleen, and kidney) ratios, which are critical criteria for reducing background signals on MRI (Figures S20–S23, Supporting Information). Additionally, cytotoxicity assay and blood parameters analysis with a clinically available dosage (0.1 mmol kg^−1^ on Gd basis) indicated no acute influence on both cellular and hematological levels (Figures S24–S35, Supporting Information).

### 2.3 MR Imaging

Based on the characteristics and potential of SMDC‐Gds, we next evaluated the *T*_1_‐weighted MR images of CT26 tumor‐bearing mice. Vertical slices on *R*_1_ distribution maps demonstrated the advantage of SMDC‐Gd_4_ over Gd‐DOTA for time‐dependent tumor accumulation and clear imaging (Figure 3d). In *R*_1_ distribution maps, the high‐intensity red signal corresponds to fatty tissues in the middle of the cross‐section, which can be attenuated or removed by fat suppression techniques in MRI. SMDC‐Gd_4_ also showed high contrast enhancement in tumor tissues, with a tumor‐to‐muscle *R*_1_ ratio (T/M) of 1.17 at 1 h after administration. This contrast enhancement was significantly greater than that of Gd‐DOTA (T/M = 0.77) (Figure 3e). The T/M value of SMDC‐Gd_4_ increased by up to 1.36 within 24 h of administration, and the tumor *R*_1_ value was 2.39 s^−1^, which was more than twice that of Gd‐DOTA (Figure 3f). PEGA‐Gd_4_ produced clear MR images, implying its utility as a polymeric contrast agent. However, its *R*_1_ appeared to be dose‐dependent and consistently remained lower than that of SMDC‐Gd_4_ within the 24 h timeframe (Figure S36, Supporting Information). The absolute quantity of Gd in tumors (Figure 3b) and the T/M ratio (Figure 3e) do not necessarily align when contrasts are evident in the blood. Given that both SMDC‐Gd_4_ and PEGA‐Gd_4_ displayed extended blood circulation times, the negligible difference in T/M ratio between SMDC‐Gd_4_ and PEGA‐Gd_4_ can likely attributed to signals emanating from muscles and capillaries, due to its prolonged half‐life in the bloodstream.

**Figure 4:**
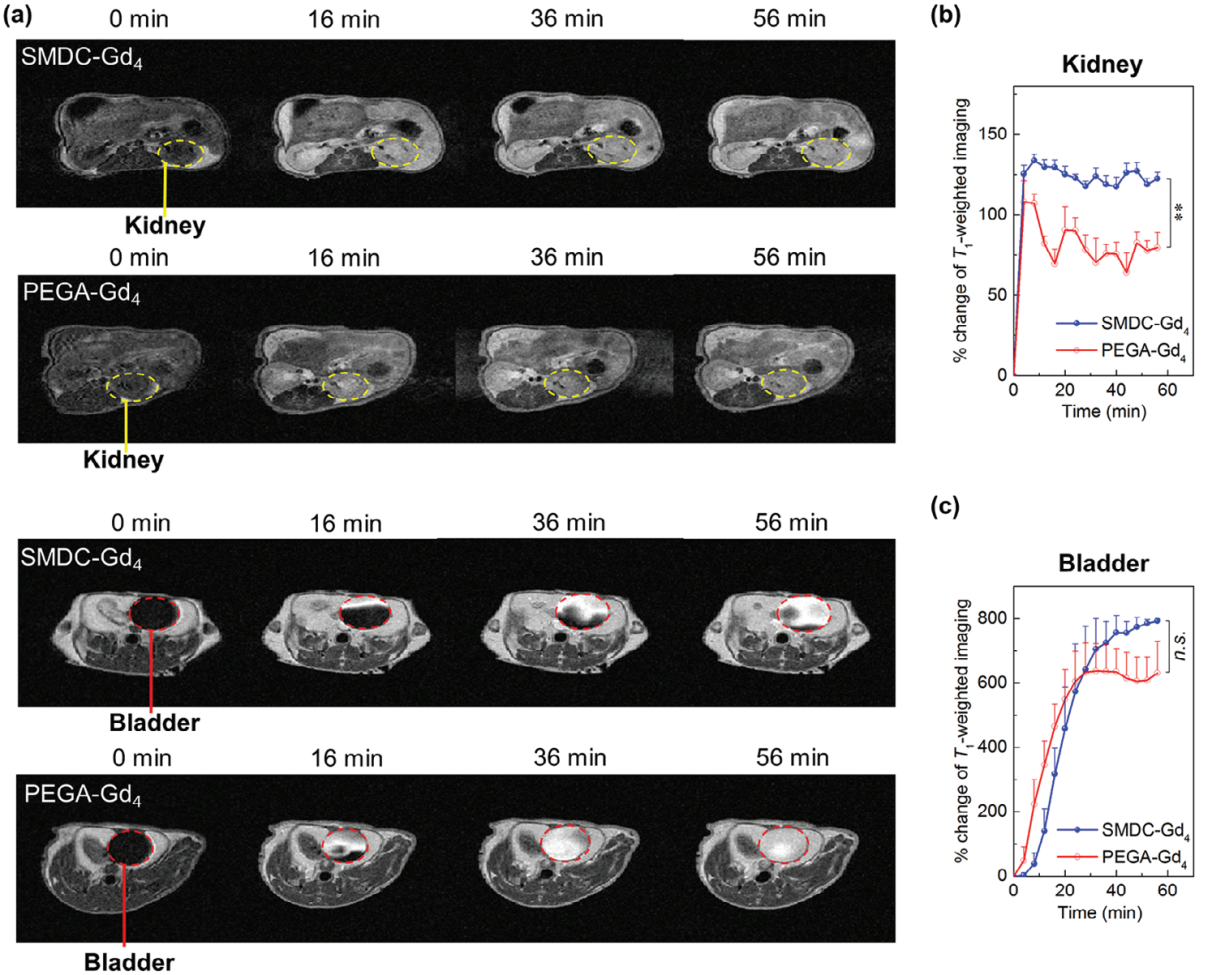
Renal excretion as measured by *T*1‐weighted MRI in tumor‐bearing mice. a) MRI of kidneys (yellow dashed areas) and bladders (red dashed areas) within 1 h after intravenous administration of SMDC‐Gd_4_ or PEGA‐Gd_4_. b,c) The comparison of longitudinal signal intensities in kidneys and bladders within 1 h after intravenous administration of SMDC‐Gd4 and PEGA‐Gd4. Data are shown as mean ± SEM, *n* = 3, ^⁎⁎^ *p* < 0.01, n.s. *p* ≥ 0.05.

Simultaneously, we monitored the MR signals in the kidneys and bladder (**Figure** **4**a). Although the accumulation profile in the kidneys and its clearance within the initial 1 h showed similar to the biodistribution results (Figure 3c), the difference in kidney and bladder clearance time indicated the permeation ability of SMDC‐Gd_4_ and PEGA‐Gd_4_ to fenestrated and negatively charged glomerular basement membrane (GBM) (Figure 4b,c). In fact, SMDC‐Gd_4_ and PEGA‐Gd_4_ had comparable surface charges; SMDC‐Gd_4_ was designed to exhibit highly dense self‐folding with a diameter of ≈6 nm, which was within the size threshold of the GBM. By contrast, PEGA‐Gd_4_ did not exhibit any SAXS signals, indicating a flexible polymer state. Therefore, even in the sub‐10 nm range, the chemical and morphological properties of single polymer strands can significantly affect the biological behavior of GBM. Note that CMC/CAC does not exist in the SMDC system. Many studies on NCAs have highlighted their imaging potential, with kidney accumulation as one of their safety parameters; there was also an influence of dissociated fractions resulting from carrier decomposition. Although further research is needed to clarify the details of GBM‐polymer filtration, our results show the validity of fine‐tuning by controlling the balance between the flexibility and rigidity of a single‐polymer design for the rapid excretion of polymer‐type CAs. Hence, optimized SMDC systems may be useful for precise and careful tumor diagnosis, even with multiple MRI injections/trials over time.

### 2.4 Gadolinium Neutron Capture Therapy

**Figure 5:**
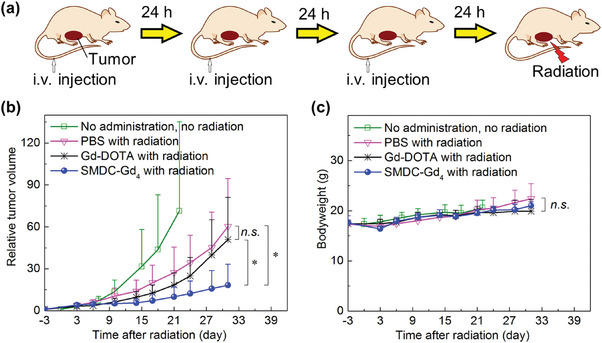
Anti‐tumor effect of SMDC‐Gd_4_ in Gd‐NCT against CT26 tumor‐bearing mice. a) Schematic illustration of the therapeutic regimen for Gd‐NCT. Daily injections for three consecutive days were given, followed by thermal neutron irradiation (for 10 min, 5MW, fluence: 2.87 × 10^12^ to 3.29 × 10^12^ thermal neutrons cm^−2^, 5.10 × 10^11^ to 5.86 × 10^11^ epithermal neutrons cm^−2^) directly toward subcutaneous solid tumors 24 h after the last injection. b) Relative tumor volumes in BALB/c mice. SMDC‐Gd_4_ combined with radiation showed a significant anti‐tumor effect compared with other groups within 31 days after radiation. Data are shown as mean ± s.d., *n* = 5, n.s. *p* ≥ 0.05, ^⁎^ *p* < 0.05. c) Bodyweight of mice. Data are shown as mean ± s.d., *n* = 5, n.s. *p* ≥ 0.05.

Gd‐based CAs are applicable for ^157^Gd‐ NCT, but have not achieved clinical success in tumor therapy.^[^ ^35^ ^]^ A potential barrier is the absence of strategy for selective delivering ^157^Gd into target tumors. Indeed, maintaining an optimal ^157^Gd‐concentration is crucial for demonstrating the desired therapeutic efficacy upon thermal neutron irradiation, with a suggested ^157^Gd‐concentration in the tumor being in the range of 50–200 ppm.^[^ ^36^, ^37^ ^]^ To reach this ^157^Gd‐concentration, high‐dosage injections were employed for Gd‐NCT. However, such strategies invariably pose risks of side effects and suboptimal therapeutic outcomes, often due to the excessive Gd fraction surrounding the tumor (shielding effect).^[^ ^36^, ^38^ ^]^ Given the clinically approved Gd complexes, a dose of 0.1–0.3 mmol kg^−1^ (based on Gd) seems practical for both MRI and Gd‐NCT applications. Yet, due to the challenges in tumor targeting, minimal success has been observed with a single injection of low molecular weight Gd complexes for Gd‐NCT. As a result, employing multiple NCA injection techniques become essential to elevate the Gd‐concentration at the tumor location. SMDC‐Gd_4_ demonstrated controllable biodistribution and clearance in a murine model of solid tumors. This motivated us to investigate ^157^Gd‐NCT using our SMDC system. To estimate the therapeutic potential, Gd concentrations in the tumors were initially evaluated by comparing the number of injections. Although a single administration of SMDC‐Gd_4_ could deliver 41.2 ppm of Gd (6.4 ppm of ^157^Gd‐basis) into the tumor 24 h after injection, three administrations (every 24 h) increased the final Gd concentration to 91.2 ppm (14.3 ppm of ^157^Gd‐basis) 24 h after the last injection (Figures S37 and S38, Supporting Information). We then investigated the feasibility of using Gd‐NCT with an SMDC system. SMDC‐Gd_4_ was tested in xenografted CT26 tumor‐bearing mice and compared with Gd‐DOTA (**Figure** **5**a). The utility of SMDC‐Gd_4_ in Gd‐NCT was evident from the suppression of tumor growth without body weight loss, whereas the Gd‐DOTA, PBS, and cold‐groups (no administration and no radiation) failed to show sufficient anti‐tumor activity (Figure 5b,c). This outcome implies that SMDC can effectively transport suitable amounts of Gd complexes into tumor tissues, enhancing the anti‐tumor impacts of NCT. Although the concentration of the ^157^Gd delivered by SMDC in this study was below the recommended range of 50–200 ppm, we still observed significant therapeutic effectiveness (Figure 5). This observation merits further examination. It is important to highlight that the benefit of drug delivery systems employing nanocarriers is not solely in the selective accumulation of therapeutic agents, but also in optimizing drug distribution within tumors. For instance, the dimensions of the nanomedicines significantly influence their extravasation and penetration into tumors, with smaller nanomedicines, such as 30 nm, presenting enhanced anti‐tumor activity compared to larger counterparts (100 nm).^[^ ^14^ ^]^ Given that the diameter of SMDC is < 10 nm, our findings likely arise from the profound tumor penetration of SMDC, facilitating the evasion of the shielding effect of thermal neutrons and ensuring efficient diffusion of electrons and *γ*‐rays post thermal neutron exposure. A similar rationale may apply to the recently introduced AGulX, an inorganic NCA with a sufficient small size (average diameter of 5 nm) to facilitate precise MR imaging and radiation therapy.^[^ ^39^, ^40^, ^41^ ^]^ Consequently, it is plausible to infer that the size of SMDC offers a distinct advantage in delivering CAs against tumors, achieving superior MR imaging and therapeutic outcomes in Gd‐NCT. While in‐depth studies are essential to refine this emerging SMDC system for drug delivery, our findings underscore the potential of fine‐tuning NCAs through self‐folding molecular design, marking a significant advancement for NCA utilization in cancer diagnosis and treatment.

## 3 Conclusion

The nanoscale environment, amount of CAs, stealth functionality, and in vivo clearance are crucial for adjusting the potential of NCAs and their selective delivery to target sites. An SMDC was designed to induce a far rare type of boosted proton longitudinal relaxivity with minimal loading of Gd complexes. Self‐folding has been shown to contribute to the formation of sub‐10 nm‐sized drug carriers and to change the nanoscale environment within a single NCA, which offers a way to obtain efficient tumor accumulation, controlled clearance, and fine tumor imaging. The utility of this SMDC system was further confirmed by the beneficial effects of thermal neutron irradiation on tumor growth inhibition. Finally, this study presents further possibilities for exploiting drug delivery using various therapeutic cargos. We are currently investigating the development of such SMDC systems.

## 4 Experimental Section

### Materials, Cell Line, and Animals

Poly (ethylene glycol) methyl ether acrylate (PEGA, *M*_n_ = 480 g mol^−1^), 2‐carboxyethyl acrylate (CEA), and 2‐(dodecylthiocarbonothioylthio)−2‐methylpropionic acid (DDMAT) were purchased from Sigma–Aldrich Corporation (St. Louis, Missouri, USA). 2,2′‐Azobis(2‐methylpropionitrile) (AIBN), *N*,*N*‐dimethylformamide (DMF), toluene, chloroform, methanol, 4‐(4,6‐dimethoxy‐1,3,5‐triazin‐2‐yl)−4‐methyl‐morpholinium chloride (DMT‐MM), gadolinium trichloride hexahydrate (GdCl_3_·6H_2_O), and phosphate‐buffered saline (PBS) were purchased from Fujifilm Wako Pure Chemical Corporation (Osaka, Japan). *s*‐2‐(4‐Aminobenzyl)−1,4,7,10‐tetraazacyclododecane *tetra*‐*tert*‐butylacetate (*p*‐NH_2_‐Bn‐DOTA‐*t*Bu) was obtained from Macrocyclics, Inc. (Plano, Texas, USA). Benzyl acrylate (BZA) and trifluoroacetic acid (TFA) were purchased from Tokyo Chemical Industry Co., Ltd. (Tokyo, Japan). Before utilization, PEGA was purified by an aluminum oxide‐packed column funnel to remove the inhibitor and degassed by five vacuum and argon backfill cycles. CEA was purified by an inhibitor remover prepacked column 30631–2 (Sigma–Aldrich Corporation, St. Louis, Missouri, USA) and degassed by five vacuum and argon backfill cycles. AIBN was purified by recrystallization and dried by vacuum. BZA, DMF, and toluene were purified by vacuum distillation. Other reagents and solvents were used as received. Murine colon carcinoma 26 (CT26) cell was purchased from ATCC (Manassas, VA, USA). The cell was subcultured in Dulbecco's Modified Eagle Medium with 10% fetal bovine serum and 1% penicillin (Merck Millipore, Burlington, MA, USA); this was maintained at 37 °C in an incubator (5% CO_2_, 95% humidified environment). All animal experiments were approved by the Animal Care and Use Committee and performed in accordance with the Guidelines for the Care and Use of Laboratory Animals set forth by Tokyo Institute of Technology, the National Institutes for Quantum Science and Technology, and Kyoto University (#: D2021008‐2).

### General Polymerization Procedure

Comb‐type random copolymers (**P1**‐**P20**) and terpolymers (**TP1**‐**TP6**) were synthesized through RAFT polymerization. Typically, a mixture of initiator (AIBN, 0.5 mg, 0.003 mmol), chain transfer agent (DDMAT, 2.2 mg, 0.006 mmol), and monomers (e.g., BZA, 48.7 mg, 0.3 mmol) in dry toluene (1 mL) of dry DMF was added into a polymerization test tube equipped with a magnetic stirring bar in a glovebox (DBO‐1.5KH‐TSO, Miwa Manufacturing Co., Ltd., Osaka, Japan). All polymerization was performed at 70 °C. Polymerization was stopped by opening the test tube and exposing it to liquid N_2_ and air. The obtained polymers were purified by dialysis against MeOH/water. Gd‐loaded copolymers (**TP4**‐**TP6**, **PEGA‐Gd_4,_** and **PEGA‐Gd_12_**) were synthesized by condensation reaction with *p*‐NH₂‐Bn‐DOTA‐*t*Bu (339.2 mg, 0.4 mmol) and DMT‐MM (132.8 mg, 0.48 mmol) in methanol/DMF mixture (20 mL, methanol:DMF = 1:1). Followed by deprotection of *t*Bu groups, chelation was performed with GdCl_3_·6H_2_O (2973.6 mg, 8.0 mmol) in Milli‐Q water. More detailed procedures were provided in the Supporting Information. The copolymerization behavior was analyzed using the Fineman–Ross method. Molecular weight, polydispersity index, DP, and conversion rate of products were characterized by nuclear magnetic resonance (NMR) and gel permeation chromatography (GPC). In detail, the ^1^H NMR spectra were recorded using a Bruker biospin AVANCE III 400A (400 MHz) (Bruker Corporation, Billerica, Massachusetts, USA) instrument with CDCl_3_, D_2_O, and DMSO‐*d*_6_ containing tetramethylsilane as the internal standard. GPC was performed at 40 °C using a JASCO Extrema HPLC system (LC‐Net II/ADC, Co‐4060, AS‐4050, PU‐4180, UV‐4070, and RI‐4030, JASCO Corporation, Tokyo, Japan) equipped with a TSKgel *α*−2500 column (linear, 7.8 mm × 300 mm; pore size, 2.5 nm; bead size, 7 µm; exclusion limit, 1 × 10^4^ g mol^−1^, Tosoh Corporation, Tokyo, Japan), a TSKgel *α*−4000 column (linear, 7.8 mm × 300 mm; pore size, 45 nm; bead size, 10 µm; exclusion limit, 1 × 10^6^ g mol^−1^, Tosoh Corporation, Tokyo, Japan), and TSKgel guardcolumn‐*α* guard column (Tosoh Corporation, Tokyo, Japan) in DMF containing lithium bromide (10 mm) at a flow rate of 1.0 mL min^−1^. Data were analyzed using JASCO ChromNAV ver. 2.04.03 (JASCO Corporation, Tokyo, Japan).

### Preparation and Characterization of SMDCs and SMDC‐Gds

SMDCs and SMDC‐Gds were prepared by adding random copolymers into Milli‐Q water, shaking for 1 h to fully dissolve, and sonicating for 1 min at room temperature (25 °C). DA was measured and calculated according to size‐exclusion chromatography coupled with multi‐angle light scattering (SEC‐MALS) results. The hydrodynamic radius (*R*_h_) was measured by dynamic light scattering (DLS). The radius of gyration (*R*_g_) was obtained by SEC‐MALS and SAXS. *ζ* potential values were measured by Zetasizer Nano ZSP. Morphology images were directly observed by TEM. In detail, SEC‐MALS was used by two solvent systems. SEC‐MALS with PBS system was performed at 40 °C using a JASCO Extrema HPLC system (LC‐Net II/ADC, Co‐4060, AS‐4050, PU‐4180, UV‐4070, and RI‐4030; JASCO Corporation, Tokyo, Japan) and DAWN 8 MALS detector (Wyatt Technology Corporation, Santa Barbara, California, USA) equipped with an OHpak SB‐804HQ column (linear, 8 mm × 300 mm; pore size, 20 nm; bead size, 10 µm; exclusion limit, 1 × 10^6^ g mol^−1^, Showa Denko K. K., Tokyo, Japan), OHpak SB‐806MHQ column (linear, 8 mm × 300 mm; pore size, 1.5 µm; bead size, 13 µm; exclusion limit, 2 × 10^7^ g mol^−1^, Showa Denko K. K., Tokyo, Japan), and OHpak SB‐G 6B guard column (Showa Denko K. K., Tokyo, Japan) at a flow rate of 1.0 mL min^−1^. SEC‐MALS with chloroform was performed at 40 °C using a TOSOH HLC‐8220 GPC system (Tosoh Corporation, Tokyo, Japan) and DAWN HELEOS II MALS detector (Wyatt Technology Corporation, Santa Barbara, California, USA) equipped with an LF‐804 column (linear, 8 mm × 300 mm; pore size, 300 nm; bead size, 6 µm; exclusion limit, 2 × 10^6^ g mol^−1^, Showa Denko K. K., Tokyo, Japan) and LF‐G guard column (Showa Denko K. K., Tokyo, Japan) at a flow rate of 1.0 mL min^−1^. Data from these SEC‐MALS systems were analyzed using ASTRA ver. 8.0.0.25 (Wyatt Technology Corporation, Santa Barbara, California, USA). DLS and *ζ* potential measurement was performed by Malvern Zetasizer Nano ZSP (Malvern Panalytical, Malvern, Worcestershire, UK) equipped with a 10 mW He–Ne laser operating at 633 nm with 173° collecting optics. Data were analyzed using Zetasizer Software ver. 7.03 (Malvern Panalytical, Malvern, Worcestershire, UK). A nano‐viewer SAXS system (Rigaku Corporation, Tokyo, Japan) was used a scattering angle was 0^o^–4^o^ at 25 °C. The results were analyzed by Smartlab Studio II ver. 4.3.239.0 (Rigaku Corporation, Tokyo, Japan); *R*_g_ values were calculated using a Guinier plot, and the SMDC formation performance was observed using a Kratky plot. TEM measurement was performed by the JEM‐2100F TEM system (JEOL, Ltd., Akishima, Tokyo). For TEM, each sample was stained on formvar/carbon‐supported copper grids (Sigma–Aldrich Corporation, ST. Louis, Missouri, USA) and dried overnight. Data were analyzed by ImageJ ver.1.53k (National Institutes of Health, Bethesda, Maryland, USA).

### Relaxivity Characterization

The longitudinal and transverse relaxation time *T*_1_ and *T*_2_ values were measured by Bruker minispec mq20 (magnetic field = 0.47 T) NMR and mq60 (magnetic field = 1.5 T) (Bruker Corporation, Billerica, Massachusetts, USA). Each sample with four concentrations (1, 0.5, 0.25, and 0.125 mm) with or without BSA (10 mg mL^−1^) was prepared prior to the measurement. Parameters of the NMR system were as follows; temperature = 37 °C, receiver gain = 80–82, recycle delay = 2. The least squares method was used for linear fitting to calculate the *r*_1_, *r*_2_, and *r*_1_/*r*_2_ values.

### Biodistribution Study

BALB/c mice (6 weeks old, female, Japan SLC Inc., Hamamatsu, Japan) with colon tumors were prepared for a biodistribution study. The mice were inoculated subcutaneously with CT26 (1 × 10^6^ cells per mouse) cells. Ten days post‐inoculation, the mice were separated into four groups (*n* = 6), intravenously injected with Gd‐DOTA, PEGA‐Gd_4_, SMDC‐Gd_4_, and SMDC‐Gd_17_ via the tail vein at 5 mg kg^−1^ of Gd. The mice were sacrificed at 1, 4, 8, 24, and 72 h after administration. Blood was collected and centrifuged to obtain the plasma. Tumors and organs, including the liver, spleen, kidney, muscle, pancreas, and brain, were excised, washed with PBS, and weighed. All samples were mixed with nitric acid (concentration = 70%, 1 mL), and acid digestion was carried out using EYELA MG‐2300 (Tokyo Rikakikai CO. LTD., Tokyo, Japan). The obtained solutions were diluted by Milli‐Q water, and the Gd concentration in each sample was measured by inductively coupled plasma mass spectrometry (ICP‐MS) using an Agilent 7700x ICP‐MS (Agilent Technologies Inc., Santa Clara, California, USA).

### MRI of CT26 Tumor‐Bearing Mice

All in vivo MRI experiments were approved by the Animal Experiment Committee of the National Institutes for Quantum Science and Technology (14‐1006‐14). BALB/c nu/nu mice (5 weeks old, female, Japan SLC Inc., Hamamatsu, Japan) were inoculated subcutaneously with CT26 (1 × 10^6^ cells per mouse) cells in Hanks′ balanced salt solution (Sigma–Aldrich, Japan). One‐week post‐inoculation, the mice were randomly separated into three groups (*n* = 4), intravenously injected with Gd‐DOTA, PEGA‐Gd_4_, and SMDC‐Gd_4_ (0.1 mmol kg^−1^ on Gd basis) via the tail vein (doses of PEGA‐Gd_4_ and SMDC‐Gd_4_ in dose‐dependence investigation were 0.1, 0.05, 0.03, and 0.01 mmol Gd kg^−1^, *n* = 2–4). Each mouse was anesthetized with isoflurane (3% for initial induction and 1%–2% during MRI scan) and placed in a prone position on a custom‐made MRI bed with a bite bar and gas mask. The respiration rate was monitored using a respiration sensor (SA Instruments, Inc., NY, USA) and regulated at 80–120 breaths min^−1^. The core body temperature was monitored using a rectal probe (FOT‐L and FTI‐10, FISO Technologies Inc., Germany) and regulated at 37.0° ± 1.0° using a water circulation pad and warm circulating air system. MRI data were acquired using a horizontal 1.0 T Bruker ICON MRI system (Bruker Biospin, Bruker Corporation, Ettlingen, Germany) with a dedicated solenoid coil for the mouse body. Following standard adjustment procedures, a pilot scan was used to accurately locate the animal's body within the magnet. Subcutaneous CT26 tumor‐bearing BALB/c nude mice were scanned before and after the intravenous injection of samples. After 24 h of injection, the mice were scanned once again. For quantitative *T*_1_ mapping, a rapid acquisition with relaxation enhancement (RARE)‐based inversion recovery sequence was used, and the following parameters were adopted: RARE factor, 4; repetition time, 10 000 ms; effective echo time, 10 ms; inversion time, 100, 300, 500, 700, 900, 1300, 1700, 1900, 2100, 2500, 2900, 3300, and 3700 ms; number of excitations, 1; slice thickness, 2 mm; field of view (FOV), 28 × 16 mm^2^; matrix size, 70 × 40; in‐plane resolution, 0.4 × 0.4 mm^2^. The fat suppression mode was off, and FOV saturation was on. The total scanning time for a single timepoint for *T*_1_ mapping was 20 min. For *T*_1_‐weighted imaging, a spin‐echo sequence was used, and the following parameters were adopted: repetition time, 400 ms; effective echo time, 7 ms; number of excitations, 4; number of slices, 20; slice thickness, 1 mm; slice gap, 1 mm; FOV, 30 × 30 mm^2^; matrix size, 150 × 150; in‐plane resolution, 0.2 × 0.2 mm^2^. The fat suppression mode was off, and FOV saturation was off. Total scanning time for a single timepoint for *T*_1_‐weighted imaging was 4 min. *T*_1_ maps and *T*_1_‐weighted images were reconstructed and analyzed using a ParaVision (Bruker) and MATLAB software (Mathworks, Natick, MA).

### Gd‐NCT of CT26 Tumor‐Bearing Mice

Colon tumor‐bearing BALB/c mice (4 weeks old, female, Japan SLC Inc., Hamamatsu, Japan) were prepared for the Gd‐NCT study. The mice were inoculated subcutaneously with CT26 (2 × 10^5^ cells per mouse) in the right thigh. The tumors were allowed to grow for about two weeks. For Gd‐NCT with one‐time injection, the mice were separated into five groups (*n* = 5). Gd‐DOTA, PEGA‐Gd_4_, and SMDC‐Gd_4_ (0.1 mmol kg^−1^ on Gd basis) were intravenously injected into the tumor‐bearing mice via the tail vein; mice in one group were used as a cold control group and cultivated without injection and radiation. Mice treated with PBS were also prepared as another control group. The inoculated mice were placed in acrylic holders, which were put on a 5‐mm‐thick thermoplastic plate that contained 40 wt.% of 6LiF (96% 6Li) to block thermal neutrons and had a circular hole in the center. The thigh with the tumor was stretched over the hole, and tumor regions were irradiated by thermal neutrons (5MW, Kyoto University Research Reactor, fluence: 2.87 × 10^12^ to 3.29 × 10^12^ thermal neutrons cm^−2^, 5.10 × 10^11^ to 5.86 × 10^11^ epithermal neutrons cm^−2^) for 10 min at 24 h after injection. For Gd‐NCT with three‐times injection, the mice were separated into three groups (*n* = 5); Gd‐DOTA, and SMDC‐Gd_4_ (0.1 mmol kg^−1^ on Gd basis) were intravenously injected to the tumor‐bearing mice via the tail vein at 0, 24, and 48 h. The PBS group was also prepared and treated with the same procedure. The mice were put on a plate as described above, and tumor regions were irradiated by thermal neutrons (5MW, Kyoto University Research Reactor, fluence: 3.27 × 10^12^ to 3.80 × 10^12^ thermal neutrons cm^−2^, 5.81 × 10^11^ to 6.10 × 10^11^ epithermal neutrons cm^−2^) for 10 min at 72 h after the first injection. Tumor growth suppression effects were evaluated in terms of tumor size (*V*), which was estimated by the following equation:

(1) $$V=a\times b^{2}/2$$

where a and b are the major and minor axes of the tumor, respectively. The statistical significance of different findings between the experimental and control groups was determined by analysis of variance with Tukey's multiple comparison test. A *p*‐value < 0.05 was considered statistically significant.

### Statistical Analysis

A two‐way analysis of variance (ANOVA) with Tukey's multiple comparisons test was employed to assess the differences in tumor mass volume between the experimental and control groups. Data analysis was conducted using GraphPad Prism version 6.00 for Windows (GraphPad Software). *P* < 0.05 was deemed statistically significant for each analysis. For all other statistical evaluations, a one‐way ANOVA with Tukey's multiple comparisons post‐hoc test was utilized, also using GraphPad Software. All data were presented as means ± s.d.

## Conflict of Interest

The authors declare no conflict of interest.

## Author Contributions

S.G. and Y.M. conceived and designed the experiments. S.G., Y.M., S.O., K.O., and Y.H. performed the polymer synthesis. S.G., S.O., and K.O. performed the material characterizations. S.G., Y.M., S.O., and M.M. performed all in vivo experiments. A.S., K.O., M.I., and I.A. performed the MRI experiments. S.G., T.N., and M.S. carried out the NCT studies. Y.M. and N.N. supervised the entire project.

## Supporting information

Supporting Information

## Data Availability

The data that support the findings of this study are available from the corresponding author upon reasonable request.
