# Supplementary material for: Self‐Folding Macromolecular Drug Carrier for Cancer Imaging and Therapy
Source: Adv Sci (Weinh). 2023 Nov 29;11(7):2304171. doi: 10.1002/advs.202304171 (PMC10870020; doi:10.1002/advs.202304171)
Supplement: Supplementary file 1 — Supporting Information [file ADVS-11-2304171-s001.pdf]

ADVANCED SCIENCE
Open Access

Supporting Information

for *Adv. Sci.*, DOI 10.1002/advs.202304171

Self-Folding Macromolecular Drug Carrier for Cancer Imaging and Therapy

Shan Gao, Yutaka Miura, Akira Sumiyoshi, Satoshi Ohno, Keisuke Ogata, Takahiro Nomoto, Makoto Matsui, Yuto Honda, Minoru Suzuki, Megumi Iiyama, Kensuke Osada, Ichio Aoki and Nobuhiro Nishiyama**

Supporting Information

Self-folding Macromolecular Drug Carrier for Cancer Imaging and Therapy

Shan Gao, Yutaka Miura, Akira Sumiyoshi, Satoshi Ohno, Keisuke Ogata, Takahiro Nomoto, Makoto Matsui, Yuto Honda, Minoru Suzuki, Megumi Iiyama, Kensuke Osada, Ichio Aoki, Nobuhiro Nishiyama**

S. Gao, Y. Miura, S. Ohno, K. Ogata, T. Nomoto, M. Matsui, Y. Honda, N. Nishiyama
Laboratory for Chemistry and Life Science, Tokyo Institute of Technology, R1-11, 4259 Nagatsuta-cho, Midori-ku, Yokohama, Kanagawa, 226-8503, Japan
E-mail: miura.y.ai@m.titech.ac.jp (Y. M.) and nishiyama.n.ad@m.titech.ac.jp (N. N.)

S. Gao, Y. Miura, S. Ohno, K. Ogata, T. Nomoto, Y. Honda, N. Nishiyama.
Department of Life Science and Technology, School of Life Science and Technology, Tokyo Institute of Technology, 4259 Nagatsuta-cho, Midori-ku, Yokohama, Kanagawa, 226-8503, Japan.

A. Sumiyoshi, M. Iiyama, K. Osada, I. Aoki.
Institute for Quantum Medical Science, National Institutes for Quantum Science and Technology, Anagawa 4-9-1, Inage, Chiba-city, Chiba, 263-8555, Japan.

T. Nomoto.
Department of Life Sciences, Graduate School of Arts and Sciences, the University of Tokyo, 3-8-1 Komaba, Meguro-ku, Tokyo 153-8902, Japan.

M. Suzuki.
Division of Particle Radiation Oncology, Particle Radiation Oncology Research Center, Institute for Integrated Radiation and Nuclear Science, Kyoto University, 2-1010, Asashiro-nishi, Kumatori-cho, Sennan-gun, Osaka 590-0494, Japan.

Contents

**1. Materials, cell lines, and animals**		S6
**2. Methods**		S6
**3. Typical polymerization procedures**		S9
**Table S1**.	List of target BZA/PEGA random copolymers with different degrees of polymerization (**P1-P20**).	S9
**Scheme S1**.	Synthesis of BZA_m-$PEGA_n$ (**P1-P20**) using reversible addition fragmentation chain transfer polymerization.	S10
**Scheme S2**.	Synthesis of BZA_m-CEA_k using reversible addition fragmentation chain transfer polymerization.	S11
**Scheme S3**.	Synthesis of $PEGA_n$-CEA_k using reversible addition fragmentation chain transfer polymerization.	S12
**Scheme S4**.	Synthesis of BZA_m-$PEGA_n$-CEA_k (**TP1-TP3**) using reversible addition fragmentation chain transfer polymerization.	S13
**Scheme S5**.	Reaction pathways for the synthesis of **TP4-TP6**.	S15
**Scheme S6**.	Reaction pathways for the synthesis of PEGA-Gd_4 and PEGA-Gd_{12}.	S17
**4. Supporting results and discussion of polymerization**		S17
**Figure S1**.	^{1}H NMR spectrum of BZA_{50}-$PEGA_{50}$ in chloroform-*d* at 25 °C.	S19
**Figure S2**.	Plot of the mole fraction of BZA in copolymers versus that in the feed to determine the reactivity ratios of BZA/PEGA copolymers (**P1-P20**).	S19
**Figure S3**.	Plots of conversion versus time in the polymerization of BZA_m-$PEGA_n$ copolymers (**P3**, **P7**, **P11**, **P15**, and **P19**).	S20
**Figure S4**.	Evolution of molecular weight with conversion for BZA_m-$PEGA_n$ polymerizations (**P3**, **P7**, **P11**, **P15**, and **P19**).	S20
**Figure S5**.	Evolution of polydispersity with conversion for BZA_m-$PEGA_n$ polymerizations (**P3**, **P7**, **P11**, **P15**, and **P19**).	S21
**Figure S6**.	^{1}H NMR spectrum of BZA_{24}-CEA_{36} in DMSO-d_6 at 25 °C	S21
**Figure S7**.	Plot of the mole fraction of BZA in copolymers versus that in the feed to determine the reactivity ratios of BZA/CEA copolymers.	S22

**Figure S8**.	^{1}H NMR spectrum of $PEGA_{100}$-CEA_{100} in chloroform-*d* at 25 °C.	S22
**Figure S9**.	Plot of the mole fraction of CEA in copolymers versus that in the feed to determine the reactivity ratios of PEGA/CEA copolymers.	S23
**Figure S10**.	^{1}H NMR spectrum of **TP1** in chloroform-*d* at 25 °C.	S23
**Figure S11**.	Plots of conversion versus time in the polymerization of **TP1-TP3**.	S24
**Figure S12**.	Evolution of molecular weight and polydispersity with conversion for **TP1-TP3** polymerizations.	S24
**Figure S13**.	^{1}H NMR spectrum of BZA_{100}-$PEGA_{90}$-$CEA_{10}(DOTA)_4$ in DMSO-d_6 at 25 °C.	S25
**Figure S14**.	^{1}H NMR spectrum of $PEGA_{124}$-$CEA_{10}(DOTA)_4$ in DMSO-d_6 at 25 °C.	S26

5. Supporting Data S27

**Table S2**.	Characterization of random copolymers **P1-P20**.	S27
**Table S3.**	Characterization of random copolymers **TP4-TP6.**	S28
**Figure S15**.	Stability and Gd-leakage from SMDC-Gds.	S28
**Figure S16**.	Time profiles of Gd concentration in the liver after intravenous injection.	S29
**Figure S17**.	Time profiles of Gd concentration in the spleen after intravenous injection.	S29
**Figure S18**.	Time profiles of Gd concentration in the brain after intravenous injection.	S30
**Figure S19**.	Time profiles of Gd concentration in the muscle after intravenous injection.	S30
**Figure S20**.	Time profiles of Gd concentration in the pancreas after intravenous injection.	S31
**Figure S21**.	Area under the Gd concentration curve (AUC) ratios of the tumor to the main organs after the intravenous administration of Gd-DOTA.	S31
**Figure S22**.	Area under the Gd concentration curve (AUC) ratios of the tumor to the main organs after the intravenous administration of PEGA-Gd_4.	S32

**Figure S23**.	Area under the Gd concentration curve (AUC) ratios of the tumor to the main organs after the intravenous administration of SMDC-Gd_4.	S32
**Figure S24**.	Area under the Gd concentration curve (AUC) ratios of the tumor to the main organs after the intravenous administration of SMDC-Gd_{17}.	S33
**Figure S25**.	Cell viability of CT26 cells treated with Gd-conjugated contrast agents.	S33
**Figure S26**.	Effects of Gd-conjugated contrast agents on the alkaline phosphatase level in mice.	S34
**Figure S27**.	Effects of Gd-conjugated contrast agents on the blood urea nitrogen in mice.	S34
**Figure S28**.	Effects of Gd-conjugated contrast agents on the creatinine level in mice.	S35
**Figure S29**.	Effects of Gd-conjugated contrast agents on the glutamic-oxaloacetic transaminase level in mice.	S35
**Figure S30**.	Effects of Gd-conjugated contrast agents on the glutamic-pyruvic transaminase level in mice.	S36
**Figure S31**.	Effects of Gd-conjugated contrast agents on the total protein level in mice.	S36
**Figure S32**.	Effects of Gd-conjugated contrast agents on the hematocrit level in mice.	S37
**Figure S33**.	Effects of Gd-conjugated contrast agents on the hemoglobin level in mice.	S37
**Figure S34**.	Effects of Gd-conjugated contrast agents on the platelet count in mice.	S38
**Figure S35**.	Effects of Gd-conjugated contrast agents on the red blood cell count in mice.	S38
**Figure S36**.	Effects of Gd-conjugated contrast agents on the white blood cell count in mice.	S39
**Figure S37**.	Contrast effect of SMDC-Gd_4 and PEGA-Gd_4 in MRI with multiple doses.	S40
**Figure S38**.	Gd concentrations in tumors and main organs 24 h after intravenous injection of SMDC-Gd_4.	S40

Figure S39. Gd concentrations in tumors and main organs 24 h after intravenous injection of Gd-DOTA. S41

.

1. Materials, cell lines and animals

Poly (ethylene glycol) methyl ether acrylate (PEGA, M_n = 480 g/mol), 2-carboxyethyl acrylate (CEA) and 2-(dodecylthiocarbonothioylthio)-2-methylpropionic acid (DDMAT) were purchased from Sigma-Aldrich Corporation (St. Louis, MO, USA). 2,2′-Azobis(2-methylpropionitrile) (AIBN), *N*,*N*-dimethylformamide (DMF), toluene, chloroform, methanol, 4-(4,6-dimethoxy-1,3,5-triazin-2-yl)-4-methyl-morpholinium chloride (DMT-MM), gadolinium trichloride hexahydrate ($GdCl_3 \cdot 6H_2O$) and phosphate-buffered saline (PBS (−)) were purchased from Fujifilm Wako Pure Chemical Corporation (Osaka, Japan). s-2-(4-Aminobenzyl)-1,4,7,10-tetraazacyclododecane tetra-*tert*-butylacetate (p-NH_2-Bn-DOTA-*t*Bu) was obtained from Macrocyclics, Inc. (Plano, TX, USA). Benzyl acrylate (BZA) and trifluoroacetic acid (TFA) were purchased from Tokyo Chemical Industry Co., Ltd. (Tokyo, Japan). Before utilization, PEGA was purified by aluminum oxide packed column funnel to remove the inhibitor and degassed by five times vacuum and argon backfill cycles. CEA was purified by the inhibitor remover prepacked column 30631-2 (Sigma-Aldrich Corporation) and degassed by five times vacuum and argon backfill cycles. AIBN was purified by recrystallisation and dried by vacuum. BZA, DMF, and toluene were purified by vacuum distillation. Other reagents and solvents were used as received. Murine colon carcinoma 26 (CT26) cells were purchased from ATCC (Manassas, VA, USA). Cells were subcultured in Dulbecco’s Modified Eagle Medium (DMEM) with 10% fetal bovine serum (FBS) and 1% penicillin (Merck Millipore, Burlington, MA, USA) and were maintained at 37 °C in an incubator (5% CO_2, 95% humidified environment). All animal experiments were approved by the Animal Care and Use Committee and performed in accordance with the Guidelines for the Care and Use of Laboratory Animals set forth by the Tokyo Institute of Technology (# D2021008-2) and National Institutes for Quantum Science and Technology.

2. Methods

2.1. Characterization and analysis methods

^{1}H nuclear magnetic resonance (NMR) spectra were recorded using a Bruker biospin AVANCE III 400A (400 MHz) (Bruker Corporation, Billerica, MA, USA) instrument with $CDCl_3$, D_2O, and DMSO-d_6 containing tetramethylsilane (TMS) as the internal standard. Gel

permeation chromatography (GPC) was performed at 40 °C using a JASCO Extrema HPLC system (LC-Net II/ADC, Co-4060, AS-4050, PU-4180, UV-4070, and RI-4030; JASCO Corporation, Tokyo, Japan) equipped with a TSKgel α-2500 column (linear, 7.8 mm × 300 mm; pore size, 2.5 nm; bead size, 7 μm; exclusion limit, 1 × 10^4 g/mol, Tosoh Corporation, Tokyo, Japan), TSKgel α-4000 column (linear, 7.8 mm × 300 mm; pore size, 45 nm; bead size, 10 μm; exclusion limit, 1 × 10^6 g/mol, Tosoh Corporation), and TSKgel guardcolumn-α guard column(Tosoh Corporation) in DMF containing lithium bromide (10 mM) at a flow rate of 1.0 mL/min. Data were analyzed using JASCO ChromNAV ver. 2.04.03 (JASCO Corporation). Size-exclusion chromatography coupled to multi-angle light scattering (SEC-MALS) was used by two solvent systems. SEC-MALS with PBS system was performed at 40 °C using a JASCO Extrema HPLC system (LC-Net II/ADC, Co-4060, AS-4050, PU-4180, UV-4070, and RI-4030; JASCO Corporation) and DAWN 8 MALS detector (Wyatt Technology Corporation, Santa Barbara, CA, USA) equipped with an OHpak SB-804HQ column (linear, 8 mm × 300 mm; pore size, 20 nm; bead size, 10 μm; exclusion limit, 1 × 10^6 g/mol; Showa Denko K. K., Tokyo, Japan), OHpak SB-806MHQ column (linear, 8 mm × 300 mm; pore size, 1.5 μm; bead size, 13 μm; exclusion limit, 2 × 10^7 g/mol; Showa Denko K. K.), and OHpak SB-G 6B guard column (Showa Denko K. K.) at a flow rate of 1.0 mL/min. SEC-MALS with chloroform was performed at 40 °C using a TOSOH HLC-8220 GPC system (Tosoh Corporation) and DAWN HELEOS II MALS detector (Wyatt Technology Corporation) equipped with an LF-804 column (linear, 8 mm × 300 mm; pore size, 300 nm; bead size, 6 μm; exclusion limit, 2 × 10^6 g/mol; Showa Denko K. K.), and an LF-G guard column (Showa Denko K. K.) at a flow rate of 1.0 mL/min. Data from these two SEC-MALS systems were analyzed using ASTRA ver. 8.0.0.25 (Wyatt Technology Corporation, Santa Barbara, CA, USA). Dynamic light scattering and ζ potential measurement was performed using Malvern Zetasizer Nano ZSP (Malvern Panalytical, Malvern, Worcestershire, UK) equipped with a 10 mW He–Ne laser operating at 633 nm with 173° collecting optics. Data were analyzed using Zetasizer Software ver. 7.03 (Malvern Panalytical). The radius of gyration (R_g) values was obtained by small angle X-ray scattering (SAXS) measurement. A nano-viewer SAXS system (Rigaku Corporation, Tokyo, Japan) was used with the scattering angle ranging from 0° to 4° at 25 °C. The results were analyzed by Smartlab Studio II ver. 4.3.239.0 (Rigaku Corporation). R_g values were calculated by the Guinier plot, and a self-folding macromolecular drug carrier (SMDC) formation performance was observed using a Kratky plot. Transmission electron microscope (TEM) measurement was performed using the JEM-2100F TEM system (JEOL, Ltd., Akishima, Tokyo). For TEM measurement, each sample was stained on formvar/carbon supported-copper grids (Sigma-

Aldrich Corporation) and dried overnight. Data were analyzed using ImageJ ver.1.53k (National Institutes of Health, Bethesda, MD, USA).

2.2. Cell viability assay

Cell viability was investigated using cell counting kit-8 (CCK-8; Dojindo Molecular Technologies Inc., Kumamoto, Japan). CT26 cells (10,000 cells/50 μL) were cultured in DMEM containing 10% FBS in a 96-well multiplate. The cells were incubated for 24 h and then exposed to Gd-DOTA, PEGA-Gd_4, SMDC-Gd_4, and SMDC-Gd_{17} in multiple concentrations (n = 5) for 48 h. The CCK-8 reagent (10 μL) was added, the cells were and then incubated for 1 h. The absorbance of media was measured at 450 nm using an iMark microplate reader (Bio-Rad Laboratories Inc., Hercules, CA, USA).

2.3. Blood parameter assay

BALB/c mice (6 weeks old, female, Japan SLC Inc., Japan) were used for biotoxicity assay. The mice were separated into four groups (n = 5). PBS (200 μL), Gd-DOTA, PEGA-Gd_4, and SMDC-Gd_4 (0.1 mmol/kg based on Gd) were intravenously injected via the tail vein. The mice were sacrificed at 4 h and 48 h after administration. Blood was collected, and 90 μL of blood was placed in a 500 μL tube containing ethylenediaminetetraacetic acid (EDTA, Dojindo Molecular Technologies Inc.) aqueous solution (10 μL, conc. = 12 mg/mL in Milli-Q water). The other blood volume was centrifuged to obtain the plasma. The whole blood with EDTA was measured using an automatic multiple blood cell counter pocH-100iV Diff (Sysmex Corporation, Kobe, Hyogo, Japan) for white blood cell (WBC), red blood cell (RBC), hemoglobin (HGB), hematocrit (HCT) and platelet (PLT) analysis. The plasma was measured by Dri-Chem 7000IZ (Fujifilm Wako Pure Chemical Corporation, Japan) for blood urea nitrogen (BUN), creatinine (CRE), glutamic-pyruvic transaminase (GPT), glutamic-oxaloacetic transaminase (GOT), alkaline phosphatase (ALP) and total protein (TP) analysis.

2.4. Biodistribution study for Gd-NCT

BALB/c mice (6 weeks old, female, Japan SLC Inc., Hamamatsu, Japan) bearing CT26 tumors were prepared. The mice were inoculated subcutaneously with CT26 (1×10^6 cells/mouse) cells. Ten days post-inoculation, mice were separated into two groups (n = 5), SMDC-Gd_4 or Gd-DOTA were injected daily intravenously for three consecutive days *via* the tail vein at the dose of 0.1 mmol/kg based on Gd for each time. The mice were sacrificed at 24 h after the last administration. Blood was collected and centrifuged to obtain the plasma. The tumor, liver and kidney were excised, washed with PBS and weighed. All samples were mixed with a nitric acid solution (concentration = 70%, 1 mL), and acid digestion was conducted using EYELA MG-2300 (Tokyo Rikakikai CO. LTD., Tokyo, Japan). Obtained solutions were

diluted by Milli-Q water, and the Gd concentration in each sample was then measured by inductively coupled plasma mass spectrometry (ICP-MS) using an Agilent 7700x ICP-MS (Agilent Technologies Inc., Santa Clara, CA, USA).

3. Typical polymerization procedures

3.1. Synthesis of BZA$_m$-PEGA$_n$ (P1-P20)

Twenty copolymers, BZA$_m$-PEGA$_n$ (**P1-P20**), with different DPs and BZA/PEGA ratios were synthesized by RAFT polymerization. Target DP, monomer feed ratios and product names are shown in **Table S1**.

Table S1. List of target BZA/PEGA random copolymers with different degrees of polymerization (**P1-P20**).

No.	Total DP$^{a)}$	BZA/PEGA$^{b)}$	Polymer
**P1**	100	2/8	BZA$_{20}$-PEGA$_{80}$
**P2**	100	4/6	BZA$_{40}$-PEGA$_{60}$
**P3**	100	5/5	BZA$_{50}$-PEGA$_{50}$
**P4**	100	6/4	BZA$_{60}$-PEGA$_{40}$
**P5**	200	2/8	BZA$_{40}$-PEGA$_{160}$
**P6**	200	4/6	BZA$_{80}$-PEGA$_{120}$
**P7**	200	5/5	BZA$_{100}$-PEGA$_{100}$
**P8**	200	6/4	BZA$_{120}$-PEGA$_{80}$
**P9**	300	2/8	BZA$_{60}$-PEGA$_{240}$
**P10**	300	4/6	BZA$_{120}$-PEGA$_{180}$
**P11**	300	5/5	BZA$_{150}$-PEGA$_{150}$
**P12**	300	6/4	BZA$_{180}$-PEGA$_{120}$
**P13**	400	2/8	BZA$_{80}$-PEGA$_{320}$
**P14**	400	4/6	BZA$_{160}$-PEGA$_{240}$
**P15**	400	5/5	BZA$_{200}$-PEGA$_{200}$
**P16**	400	6/4	BZA$_{240}$-PEGA$_{160}$
**P17**	500	2/8	BZA$_{100}$-PEGA$_{400}$
**P18**	500	4/6	BZA$_{200}$-PEGA$_{300}$
**P19**	500	5/5	BZA$_{250}$-PEGA$_{250}$
**P20**	500	6/4	BZA$_{300}$-PEGA$_{200}$

[a] Total degree of polymerization (DP); [b] Feed ratio of the BZA monomer to the PEGA monomer in the design.

The typical polymerization procedure was described as follows: For the synthesis of BZA_{50}-$PEGA_{50}$ (**P3**), all manipulations of the air-sensitive materials were performed with the rigorous exclusion of oxygen and moisture in flame- or oven-dried glassware on a high vacuum-line or in an argon-filled gas recycle purification system equipped a glovebox (DBO-1.5KH-TSO, Miwa Manufacturing Co., Ltd., Osaka, Japan). The synthetic route is shown in **Scheme S1**. A mixture of AIBN (0.5 mg, 0.003 mmol) and DDMAT (2.2 mg, 0.006 mmol) in dry toluene (1 mL) was added into the polymerization test tube equipped with a magnetic stirring bar in the glovebox. BZA (48.7 mg, 0.3 mmol) and PEGA (144.0 mg, 0.3 mmol) were added into the test tube, and polymerization was then started in a 70 °C oil bath and continued for 16 h. The polymerization reaction was terminated by liquid nitrogen. The conversion was confirmed by ^{1}H NMR. The product was purified by dialysis (Spectra/Por dialysis membrane, MWCO of 6–8 kD) against methanol and Milli-Q water. After lyophilization, the obtained BZA_{50}-$PEGA_{50}$ was analyzed by ^{1}H NMR (**Figure S1**), GPC and SEC-MALS. ^{1}H NMR ($CDCl_3$ with 0.03% TMS, 400 MHz): δ (ppm) = 0.88 (*t*, 3H, -S-CH_2-$(CH_2)_{10}$-$C\underline{H}_3$), 1.10-1.29 (*m*, 20H, -S-CH_2-$(C\underline{H}_2)_{10}$-CH_3), 1.33-1.99 (*m*, 206H, -($C\underline{H}_2$-CH(BZA segment))$_{50}$-, -($C\underline{H}_2$-CH(PEGA segment))$_{50}$-, $(C\underline{H}_3)_2$-C(COOH)-), 2.19-2.53 (*m*, 100H, -(CH_2-$C\underline{H}$(BZA segment))$_{50}$-, -(CH_2-$C\underline{H}$(PEGA segment))$_{50}$-), 3.31-3.40 (*m*, 152H, -CH_2-CH_2-O-(CH_2-CH_2-O)$_8$-$C\underline{H}_3$, -S-$C\underline{H}_2$-$(CH_2)_{10}$-CH_3), 3.42-3.74 (*m*, 1700H, -CH_2-$C\underline{H}_2$-O-($C\underline{H}_2$-$C\underline{H}_2$-O)$_8$-CH_3), 3.90-4.35 (*m*, 100H, -$C\underline{H}_2$-CH_2-O-(CH_2-CH_2-O)$_8$-CH_3), 4.81-5.16 (*m*, 100H, -COO-$C\underline{H}_2$-C_6H_5), 7.17-7.40 (*m*, 250H, -COO-CH_2-$C_6\underline{H}_5$). Conv.,$_{BZA}$ = 96.0 %, Conv.,$_{PEGA}$ = 92.0 %, $M_{n,NMR}$ = 30,200 g/mol, M_w/M_n = 1.82.

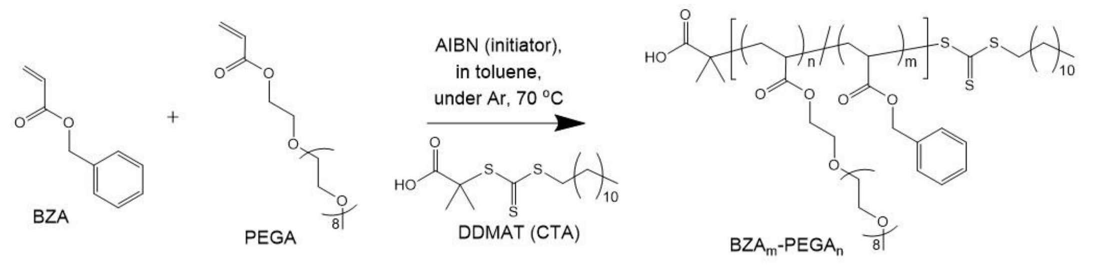

Scheme S1. Synthesis of BZA_m-$PEGA_n$ (**P1-P20**) using reversible addition fragmentation chain transfer polymerization.

3.2. Synthesis of BZA_m-CEA_k

The procedure of BZA_{24}-CEA_{36} synthesis is shown in **Scheme S2**. A mixture of AIBN (0.5 mg, 0.003 mmol) and DDMAT (2.2 mg, 0.006 mmol) in dry DMF (1 mL) was added into the polymerization test tube equipped with a magnetic stirring bar in the glovebox. BZA (38.9 mg, 0.24 mmol) and CEA (51.9 mg, 0.36 mmol) were added into the test tube, and polymerization was then started in a 70 °C oil bath and continued for 20 min. The polymerization reaction was terminated by liquid nitrogen. The product was purified by dialysis (Spectra/Por dialysis membrane, MWCO of 3.5 kD) against methanol and Milli-Q water. After lyophilization, the obtained BZA_{40}-CEA_{60} and conversion were analyzed by ^{1}H NMR (**Figure S6**). ^{1}H NMR (DMSO-d_6 with 0.03% TMS, 400 MHz): δ (ppm) = 0.88 (*t*, 3H, -S-CH_2-$(CH_2)_{10}$-C<u>*H*</u>$_3$), 1.01-1.29 (*m*, 20H, -S-CH_2-(C<u>*H*</u>$_2$)$_{10}$-CH_3), 1.33-1.99 (*m*, 126H, -(C<u>*H*</u>$_2$-CH(BZA segment))$_m$-, -(C<u>*H*</u>$_2$-CH(CEA segment))$_k$-, (C<u>*H*</u>$_3$)$_2$-C(COOH)-), 2.18-2.47 (*m*, 60H, -(CH_2-C<u>*H*</u>(BZA segment))$_m$-, -(CH_2-C<u>*H*</u>(CEA segment))$_k$-), 2.49-2.75 (*m*, 72H, -COO-CH_2-C<u>*H*</u>$_2$-COOH), 3.30 (*d*, 2H, -S-C<u>*H*</u>$_2$-$(CH_2)_{10}$-CH_3), 3.80-4.60 (*m*, 72H, -COO-C<u>*H*</u>$_2$-CH_2-COOH), 4.72-5.19 (*m*, 48H, -COO-C<u>*H*</u>$_2$-C_6H_5), 7.08-7.38 (*m*, 120H, -COO-CH_2-C_6<u>*H*</u>$_5$). Conv.,$_{BZA}$ = 64.1 %, Conv.,$_{CEA}$ = 61.0 %, $M_{n,NMR}$ = 9,600 g/mol.

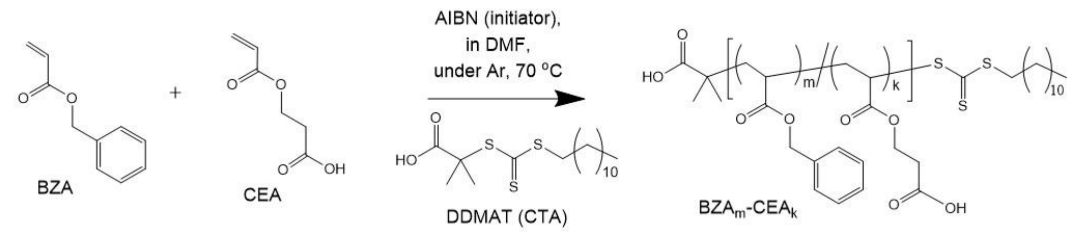

Scheme S2. Synthesis of BZA_m-CEA_k using reversible addition fragmentation chain transfer polymerization.

3.3. Synthesis of $PEGA_n$-CEA_k

The procedure of $PEGA_{100}$-CEA_{100} synthesis is shown in **Scheme S3**. A mixture of AIBN (0.5 mg, 0.003 mmol) and DDMAT (2.2 mg, 0.006 mmol) in dry DMF (1 mL) was added into the polymerization test tube equipped with a magnetic stirring bar in the glovebox. PEGA (432.0 mg, 0.90 mmol) and CEA (129.7 mg, 0.90 mmol) were added into the test tube, and polymerization was then started in a 70 °C oil bath and continued for 20 min. The polymerization reaction was terminated by liquid nitrogen. The product was purified by dialysis (Spectra/Por dialysis membrane, MWCO of 6–8 kD) against methanol and Milli-Q water. After lyophilization, the obtained $PEGA_{100}$-CEA_{100} and conversion were analyzed by ^{1}H NMR (**Figure S8**). ^{1}H NMR (DMSO-d_6 with 0.03% TMS, 400 MHz): δ (ppm) = 0.88 (*t*, 3H, -S-CH_2-$(CH_2)_{10}$-C<u>*H*</u>$_3$), 1.07-1.31 (*m*, 20H, -S-CH_2-(C<u>*H*</u>$_2$)$_{10}$-CH_3), 1.33-2.02 (*m*, 406H, -(C<u>*H*</u>$_2$-

CH(PEGA segment))$_n$-, -(C$\underline{H}_2$-CH(CEA segment))$_k$-, (C$\underline{H}_3$)$_2$-C(COOH)-), 2.08-2.58 (*m*, 200H, -(CH$_2$-C$\underline{H}$(PEGA segment))$_n$-, -(CH$_2$-C$\underline{H}$(CEA segment))$_k$-), 2.59-2.77 (*m*, 200H, -COO-CH$_2$-C$\underline{H}_2$-COOH), 3.31-3.40 (*m*, 302H, -CH$_2$-CH$_2$-O-(CH$_2$-CH$_2$-O)$_8$-C$\underline{H}_3$, -S-C$\underline{H}_2$-(CH$_2$)$_{10}$-CH$_3$), 3.42-3.74 (*m*, 3400H, -CH$_2$-C$\underline{H}_2$-O-(C$\underline{H}_2$-C$\underline{H}_2$-O)$_8$-CH$_3$), 3.98-4.47 (*m*, 400H, -C$\underline{H}_2$-CH$_2$-O-(CH$_2$-CH$_2$-O)$_8$-CH$_3$, -COO-C$\underline{H}_2$-CH$_2$-COOH). Conv.,$_{PEGA}$ = 70.1 %, Conv.,$_{CEA}$ = 69.7 %, $M_{n,NMR}$ = 64,400 g/mol.

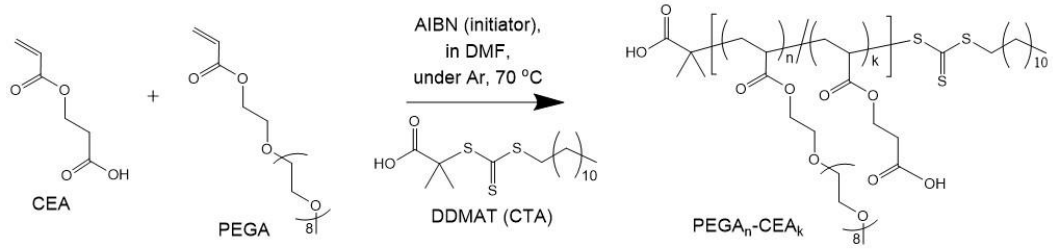

Scheme S3. Synthesis of PEGA$_n$-CEA$_k$ using reversible addition fragmentation chain transfer polymerization.

3.4. Synthesis of BZA$_m$-PEGA$_n$-CEA$_k$ (TP1-TP3)

The terpolymers, *i.e.*, BZA$_{100}$-PEGA$_{90}$-CEA$_{10}$ (**TP1**), BZA$_{100}$-PEGA$_{80}$-CEA$_{20}$ (**TP2**) and BZA$_{100}$-PEGA$_{70}$-CEA$_{30}$ (**TP3**), were prepared to introduce Gd complexes and form the SMDC in aqueous solution. The typical procedure for the synthesis of **TP1** is shown in **Scheme S4**. A mixture of AIBN (0.5 mg, 0.003 mmol) and DDMAT (2.2 mg, 0.006 mmol) in dry DMF (1 mL) was added into the polymerization test tube equipped with a magnetic stirring bar in the glovebox. BZA (146.0 mg, 0.90 mmol), PEGA (388.8 mg, 0.81 mmol) and CEA (13.0 mg, 0.09 mmol) were added into the test tube, and polymerization was then started in a 70 °C oil bath and continued for 20 min. The polymerization reaction was terminated by liquid nitrogen. The product was purified by dialysis (Spectra/Por dialysis membrane, MWCO of 6–8 kD) against methanol and Milli-Q water. After lyophilization, the obtained BZA$_{100}$-PEGA$_{90}$-CEA$_{10}$ and conversion were analyzed by ^{1}H NMR (**Figure S10**), GPC and SEC-MALS. ^{1}H NMR (DMSO-d_6 with 0.03% TMS, 400 MHz): δ (ppm) = 0.88 (*t*, 3H, -S-CH$_2$-(CH$_2$)$_{10}$-C$\underline{H}_3$), 1.10-1.29 (*m*, 20H, -S-CH$_2$-(C$\underline{H}_2$)$_{10}$-CH$_3$), 1.33-1.99 (*m*, 406H, -(C$\underline{H}_2$-CH(BZA segment))$_m$-, -(C$\underline{H}_2$-CH(PEGA segment))$_n$-, -(C$\underline{H}_2$-CH(CEA segment))$_k$-, (C$\underline{H}_3$)$_2$-C(COOH)-), 2.19-2.53 (*m*, 200H, -(CH$_2$-C$\underline{H}$(BZA segment))$_m$-, -(CH$_2$-C$\underline{H}$(PEGA segment))$_n$-, -(CH$_2$-C$\underline{H}$(CEA segment))$_k$-), 2.60-2.75 (*m*, 20H, -COO-CH$_2$-C$\underline{H}_2$-COOH), 3.31-3.40 (*m*, 272H, -CH$_2$-CH$_2$-O-(CH$_2$-CH$_2$-O)$_8$-C$\underline{H}_3$, -S-C$\underline{H}_2$-(CH$_2$)$_{10}$-CH$_3$), 3.42-3.74 (*m*, 3060H, -CH$_2$-C$\underline{H}_2$-O-(C$\underline{H}_2$-C$\underline{H}_2$-O)$_8$-CH$_3$), 3.90-4.40 (*m*, 200H, -C$\underline{H}_2$-CH$_2$-O-(CH$_2$-CH$_2$-O)$_8$-CH$_3$, -COO-C$\underline{H}_2$-CH$_2$-COOH), 4.81-5.16 (*m*,

200H, -COO-C$\underline{H}_2$-C_6H_5), 7.17-7.40 (*m*, 500H, -COO-CH_2-$C_6\underline{H}_5$). Conv.,$_{BZA}$ = 73.3 %, Conv.,$_{PEGA}$ = 65.7 %, Conv.,$_{CEA}$ = 57.1 %, $M_{n,NMR}$ = 62,700 g/mol, M_w/M_n = 1.62.

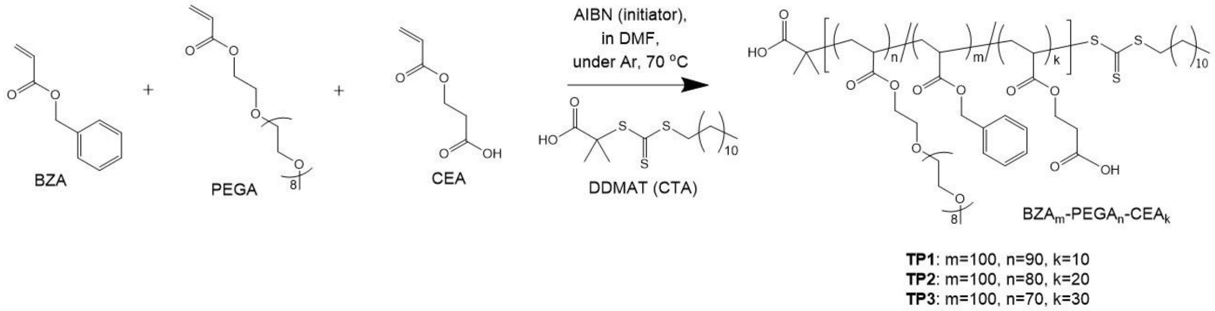

Scheme S4. Synthesis of BZA_m-$PEGA_n$-CEA_k (**TP1-TP3**) using reversible addition fragmentation chain transfer polymerization.

3.5. Synthesis of BZA_m-$PEGA_n$-CEA_k(Gd-DOTA)$_j$ (TP4-TP6)

Gd loaded terpolymers, BZA_{100}-$PEGA_{90}$-CEA_{10}(Gd-DOTA)$_4$ (**TP4**), BZA_{100}-$PEGA_{80}$-CEA_{20}(Gd-DOTA)$_9$ (**TP5**) and BZA_{100}-$PEGA_{70}$-CEA_{30}(Gd-DOTA)$_{17}$ (**TP6**) were synthesized to prepare SMDC-Gds in aqueous solutions according to the following typical polymerization procedure of **TP4**: in detail, **TP4** for SMDC-Gd_4, **TP5** for SMDC-Gd_9, and **TP6** for SMDC-Gd_{17}.

The typical synthetic route of **TP4** for preparing SMDC-Gd_4 is shown in **Scheme S5**. In the first step, *p*-NH_2-Bn-DOTA-*t*Bu was conjugated to the CEA segment in random terpolymer **TP1** by a condensation reaction. A mixture of **TP1** (2449 mg, 0.04 mmol) and DMT-MM (132.8 mg, 0.48 mmol) in methanol/DMF mixture (20 mL, methanol:DMF = 1:1) was added into a mighty vial equipped with a magnetic stirring bar and stirred for 1 h at 0 °C. Subsequently, *p*-NH_2-Bn-DOTA-*t*Bu (339.2 mg, 0.4 mmol) was added in the solution, and the reaction continued overnight at room temperature. The product was purified by dialysis (Spectra/Por dialysis membrane, MWCO of 6‑8 kD) against methanol and Milli-Q water. Then, BZA_{100}-$PEGA_{90}$-CEA_{10}(DOTA-*t*Bu)$_4$ was obtained by lyophilization.

In the second synthesis step of **TP4**, DOTA-*t*Bu segments were deprotected by adding all the purified BZA_{100}-$PEGA_{90}$-CEA_{10}(DOTA-*t*Bu)$_4$ in the TFA/chloroform mixture (20 mL, TFA:chloroform = 1:1), vigorously stirred in a mighty vial overnight at room temperature. The product was purified by dialysis (Spectra/Por dialysis membrane, MWCO of 6‑8 kD) against methanol and Milli-Q water. And then the BZA_{100}-$PEGA_{90}$-CEA_{10}(DOTA)$_4$ was obtained by lyophilization and analyzed by ^{1}H NMR (**Figure S13**). ^{1}H NMR (DMSO-d_6 with TMS, 400 MHz): δ(ppm) = 0.88 (*t*, 3H, -S-CH_2-$(CH_2)_{10}$-C$\underline{H}_3$), 0.93-1.27 (*m*, 20H, -S-CH_2-(C$\underline{H}_2$)$_{10}$-CH_3),

1.30-1.98 (*m*, 406H, -(C*H*$_2$-CH(BZA segment))$_m$-, -(C*H*$_2$-CH(PEGA segment))$_n$-, -(C*H*$_2$-CH(CEA segment))$_k$-, (C*H*$_3$)$_2$-C(COOH)-), 2.05-2.43 (*m*, 200H, -(CH$_2$-C*H*(BZA segment))$_m$-, -(CH$_2$-C*H*(PEGA segment))$_n$-, -(CH$_2$-C*H*(CEA segment))$_k$-), 2.54-2.68 (*m*, 20H, -COO-CH$_2$-C*H*$_2$-COOH, -COO-CH$_2$-C*H*$_2$-CO-DOTA segment), 3.18-3.27 (*m*, 422H, -CH$_2$-CH$_2$-O-(CH$_2$-CH$_2$-O)$_8$-C*H*$_3$, -C*H*$_2$-C*H*$_2$-N(CH$_2$COOH)-, -S-C*H*$_2$-(CH$_2$)$_{10}$-CH$_3$), 3.28-3.71 (*m*, 3100H, -CH$_2$-C*H*$_2$-O-(C*H*$_2$-C*H*$_2$-O)$_8$-CH$_3$, -CH$_2$-CH$_2$-N(C*H*$_2$COOH)-, -NH-C$_6$H$_4$-C*H*$_2$-), 3.85-4.35 (*m*, 200H, -C*H*$_2$-CH$_2$-O-(CH$_2$-CH$_2$-O)$_8$-CH$_3$, -COO-C*H*$_2$-CH$_2$-COOH, -COO-C*H*$_2$-CH$_2$-CO-DOTA segment), 4.77-5.15 (*m*, 200H, -COO-C*H*$_2$-C$_6$H$_5$), 7.00-7.60 (*m*, 540H, -COO-CH$_2$-C$_6$*H*$_5$, -NH-C$_6$*H*$_4$-CH$_2$-).

In the final synthesis step of **TP4**, Gd was chelated to the DOTA ligand. A mixture of all the deprotected BZA$_{100}$-PEGA$_{90}$-CEA$_{10}$(DOTA)$_4$ and $GdCl_3 \cdot 6H_2O$ (2973.6 mg, 8.0 mmol) in Milli-Q water was added into a mighty vial equipped with a magnetic stirring bar. The chelation reaction was started in a 60 °C water bath and continued overnight. To remove Gd that was not chelated to DOTA but reacted with the residual carboxyl group in CEA segments, the product was subjected to dialysis (Spectra/Por dialysis membrane, MWCO of 6–8 kD) against Milli-Q water, mixed with PBS (−) (100 mL) and vigorously stirred overnight at room temperature. The product was purified by filtration (syringe-driven filter unit) and dialysis (Spectra/Por dialysis membrane, MWCO of 6–8 kD) against Milli-Q water. Then the **TP4** was obtained by lyophilization. The amount of Gd loaded into each polymer was confirmed by ICP-MS.

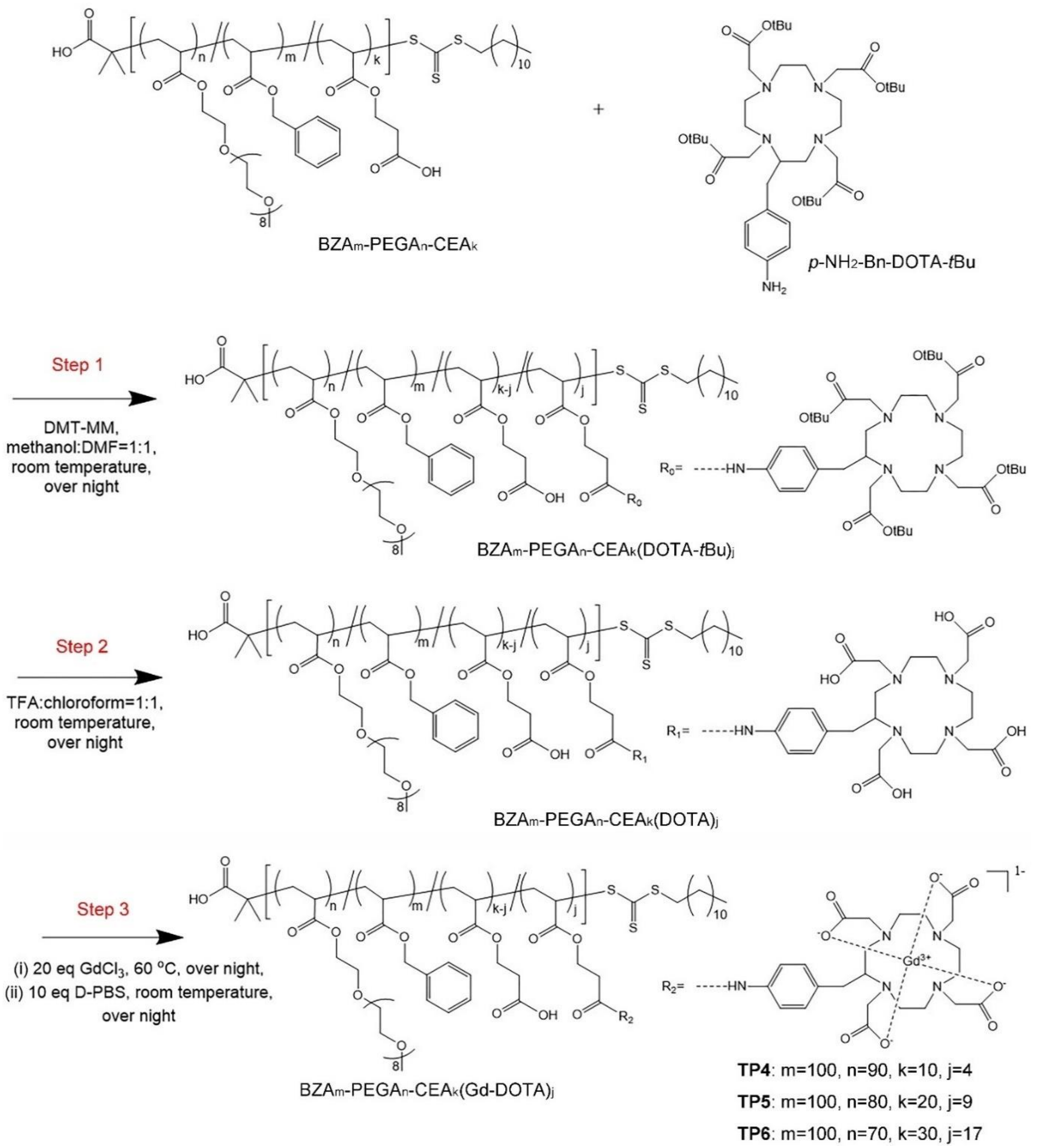

Scheme S5. Reaction pathways for the synthesis of **TP4-TP6**.

3.6. Synthesis of PEGA$_n$-CEA$_k$(Gd-DOTA)$_j$ (PEGA-Gd$_4$ and PEGA-Gd$_{12}$)

Utilized as polymer control groups, PEGA$_{124}$-CEA$_{10}$(Gd-DOTA)$_4$, named PEGA-Gd$_4$, and PEGA$_{104}$-CEA$_{30}$(Gd-DOTA)$_{12}$, named PEGA-Gd$_{12}$, were polymerized by the following typical polymerization procedure of PEGA-Gd$_4$. These two copolymers only contained hydrophilic segments, where were expected to be dissolved in water as polymer chains without construct particles.

The typical synthetic route of PEGA-Gd_4 is shown in **Scheme S6**. In the first step, a mixture of $PEGA_{124}$-CEA_{10} (2452 mg, 0.04 mmol) and DMT-MM (132.8 mg, 0.48 mmol) in a methanol/DMF mixture (20 mL, methanol:DMF = 1:1) was added into a mighty vial equipped with a magnetic stirring bar and stirred for 1 h at 0 °C. Subsequently, *p*-NH_2-Bn-DOTA-*t*Bu (339.2 mg, 0.4 mmol) was added in the solution, and the reaction continued overnight at room temperature. The product was purified by dialysis (Spectra/Por dialysis membrane, MWCO of 6 ‒ 8 kD) against methanol and Milli-Q water. Then, $PEGA_{124}$-CEA_{10}(DOTA-*t*Bu)$_4$ was obtained by lyophilization.

In the second synthesis step of PEGA-Gd_4, DOTA-*t*Bu segments were deprotected by adding all the purified $PEGA_{124}$-CEA_{10}(DOTA-*t*Bu)$_4$ in the TFA/chloroform mixture (20 mL, TFA:chloroform = 1:1), vigorously stirred in a mighty vial overnight at room temperature. The product was purified by dialysis (Spectra/Por dialysis membrane, MWCO of 6‒8 kD) against methanol and Milli-Q water. Then, the $PEGA_{124}$-CEA_{10}(DOTA)$_4$ was obtained by lyophilization and analyzed by ^{1}H NMR (**Figure S14**). ^{1}H NMR (DMSO-d_6 with TMS, 400 MHz): δ (ppm) = 0.88 (*t*, 3H, -S-CH_2-$(CH_2)_{10}$-C$\underline{H}_3$), 0.93-1.27 (*m*, 20H, -S-CH_2-(C$\underline{H}_2$)$_{10}$-CH_3), 1.30-1.98 (*m*, 274H, -(C$\underline{H}_2$-CH(PEGA segment))$_n$-, -(C$\underline{H}_2$-CH(CEA segment))$_k$-, (C$\underline{H}_3$)$_2$-C(COOH)-), 2.05-2.43 (*m*, 134H, -(CH_2-C$\underline{H}$(PEGA segment))$_n$-, -(CH_2-C$\underline{H}$(CEA segment))$_k$-), 2.54-2.68 (*m*, 20H, -COO-CH_2-C$\underline{H}_2$-COOH, -COO-CH_2-C$\underline{H}_2$-CO-DOTA segment), 3.18-3.27 (*m*, 524H, -CH_2-CH_2-O-(CH_2-CH_2-O)$_8$-C$\underline{H}_3$, -C$\underline{H}_2$-C$\underline{H}_2$-N(CH_2COOH)-, -S-C$\underline{H}_2$-$(CH_2)_{10}$-CH_3), 3.28-3.71 (*m*, 4256H, -CH_2-C$\underline{H}_2$-O-(C$\underline{H}_2$-C$\underline{H}_2$-O)$_8$-CH_3, -CH_2-CH_2-N(C$\underline{H}_2$COOH)-, -NH-C_6H_4-C$\underline{H}_2$-), 3.85-4.35 (*m*, 268H, -C$\underline{H}_2$-CH_2-O-(CH_2-CH_2-O)$_8$-CH_3, -COO-C$\underline{H}_2$-CH_2-COOH, -COO-C$\underline{H}_2$-CH_2-CO-DOTA segment), 7.00-7.60 (*m*, 40H, -NH-$C_6\underline{H}_4$-CH_2-).

In the final synthesis step of PEGA-Gd_4, Gd was chelated to the DOTA ligand. A mixture of all the deprotected $PEGA_{124}$-CEA_{10}(DOTA)$_4$ and $GdCl_3{\cdot}6H_2O$ (2973.6 mg, 8.0 mmol) in Milli-Q water was added into a mighty vial equipped with a magnetic stirring bar. The chelation reaction was started in a 60 °C water bath and continued overnight. To remove Gd that was not chelated to DOTA but reacted with the residual carboxyl group in CEA segments, the product was subjected to dialysis (Spectra/Por dialysis membrane, MWCO of 6-8 kD) against Milli-Q water, mixed with PBS (−) (100 mL) and kept vigorously stirring overnight at room temperature. The product was purified by filtration (syringe-driven filter unit) and dialysis (Spectra/Por dialysis membrane, MWCO of 6-8 kD) against Milli-Q water. And then the PEGA-Gd_4 was obtained by lyophilization. The amount of loaded-Gd in each polymer was confirmed by ICP-MS.

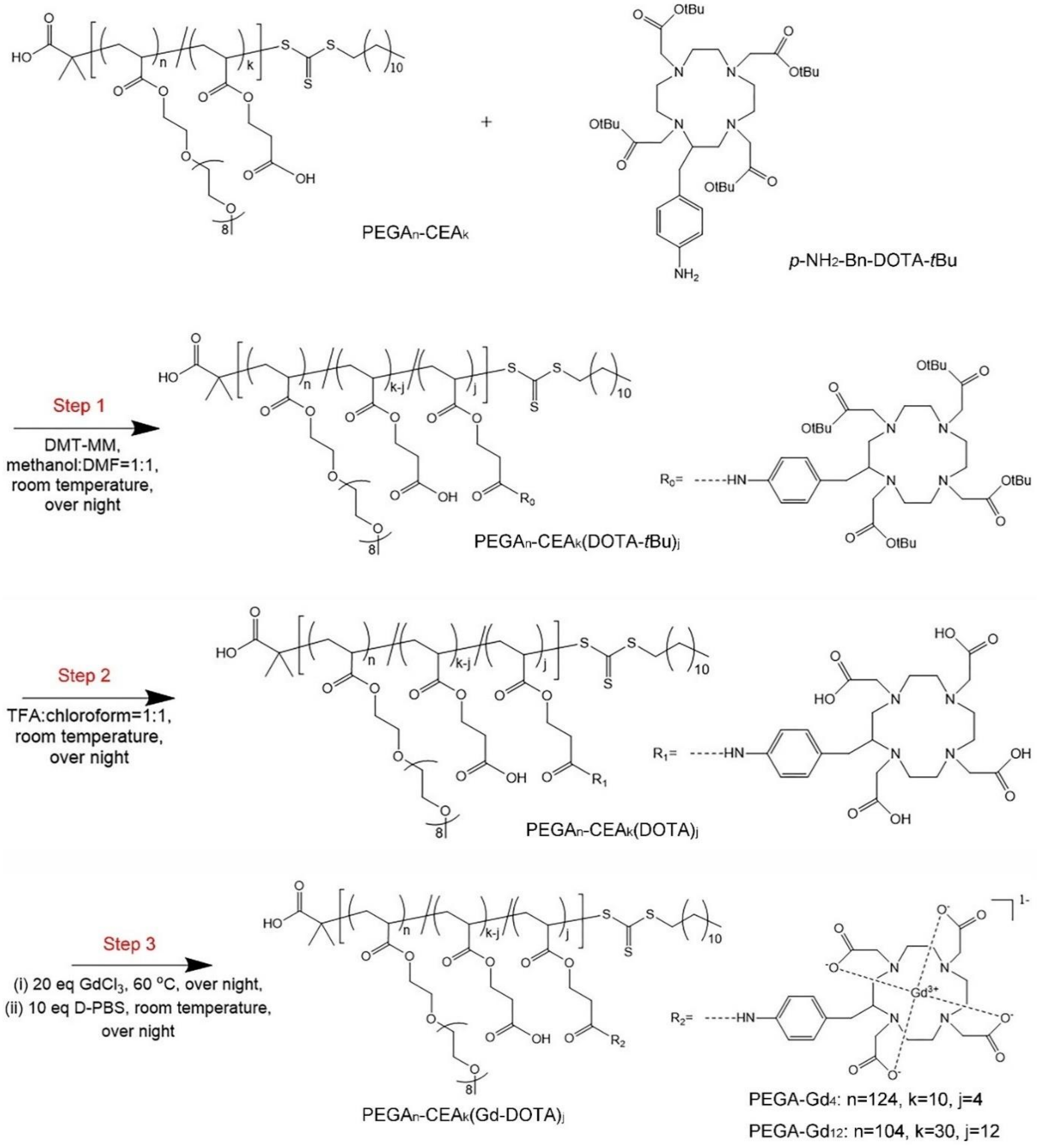

Scheme S6. Reaction pathways for the synthesis of PEGA-Gd_4 and PEGA-Gd_{12}.

4. Supporting results and discussion of polymerization

To achieve uniform SMDC formation, the randomness of the polymer sequence in the base polymer and living nature of polymerization are important. Thus, we confirm the polymerization behaviors and reactivity ratios of BZA/PEGA, BZA/CEA, and PEGA/CEA systems. First, four copolymers, BZA_m-$PEGA_n$, were synthesized according to the polymerization procedure described above "(1) Synthesis of BZA_m-$PEGA_n$ (**P1**-**P20**)" excluding the monomer feed ratio (BZA/PEGA = 2/8, 4/6, 6/4 and 8/2) and the polymerization

time (t = 20 min). The mole fractions of monomers (M_a and M_b) in feed and copolymer were obtained by ^{1}H NMR (e.g., **Figure S1**), where the BZA and PEGA monomers were defined as M_a and M_b, respectively. Generally, the reactivity ratio r_a is a measure of the tendency toward self-propagation of M_a-chain ends to add additional M_a. Likewise, the reactivity ratio for the M_b-chain end is given by r_b. The Fineman-Ross method was used for data analysis to generate and fit reactivity ratios. The obtained reactivity ratios of BZA/PEGA copolymerization are shown in **Figure S2**; r_a was equal to 1.28 and r_b was equal to 0.73. As both r_a and r_b were close to 1.0, it is reasonable to conclude that the obtained copolymer had a totally random sequence. The kinetic analysis of BZA/PEGA copolymerization (**Figures S3**–**S5**, where **P3**, **P7**, **P11**, **P15** and **P19** were selected as examples) revealed that these polymerizations were preceded by following living nature, however, the polydispersity value increased over 70% conversion because of the increase in viscosity. The same analysis was adopted to the BZA/CEA and PEGA/CEA copolymerization, and all indicated good randomness of sequence (**Figures S6**–**S9**). **TP1-TP3** preparations were also evaluated by kinetic plots using total conversion versus M_n/polymerization time. As shown in **Figures S10**–**S12**, these reactions also showed controlled/living polymerization behaviors with relatively narrow polydispersity when their conversion values were <70%. Because the randomness of the polymer sequence was evidenced by analysis of BZA/PEGA, BZA/CEA, and PEGA/CEA copolymerization, all polymers, BZA_m-$PEGA_n$-CEA_k, might be inferred as terpolymers equipped with a random order of monomer units. Overall, we precisely analyzed all polymerization reactions and determined an appropriate condition for preparing terpolymers; thus, we used these polymers as platforms for further synthesis, analysis, and characterization in this study.

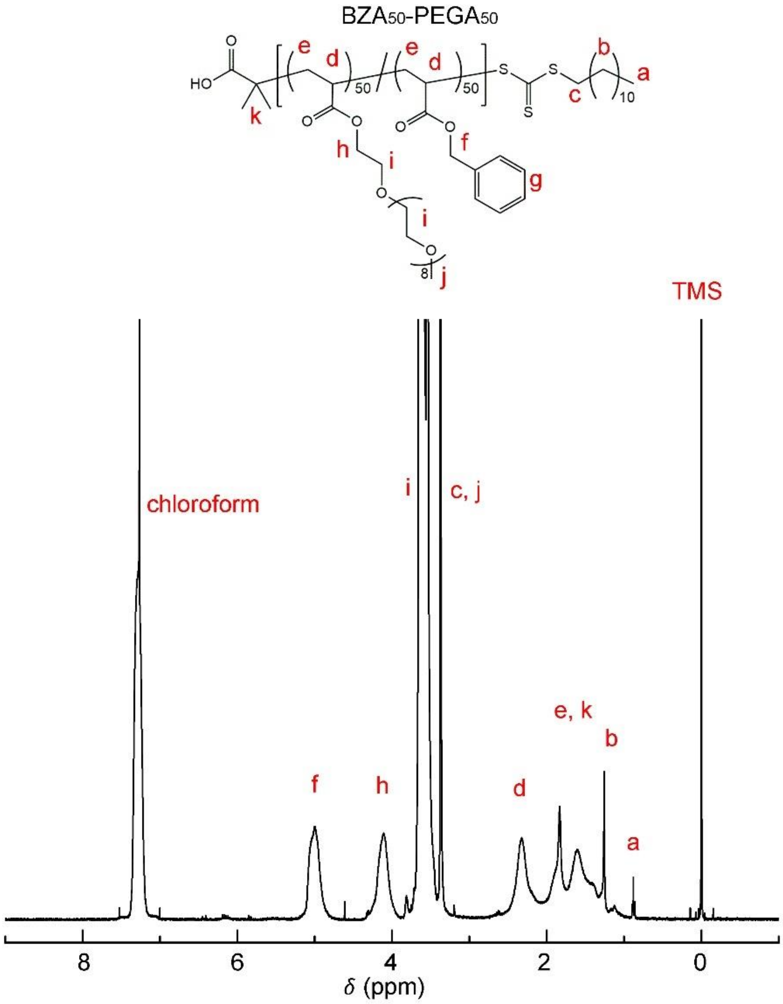

Figure S1. ^{1}H NMR spectrum of BZA_{50}-$PEGA_{50}$ in chloroform-*d* at 25 °C.

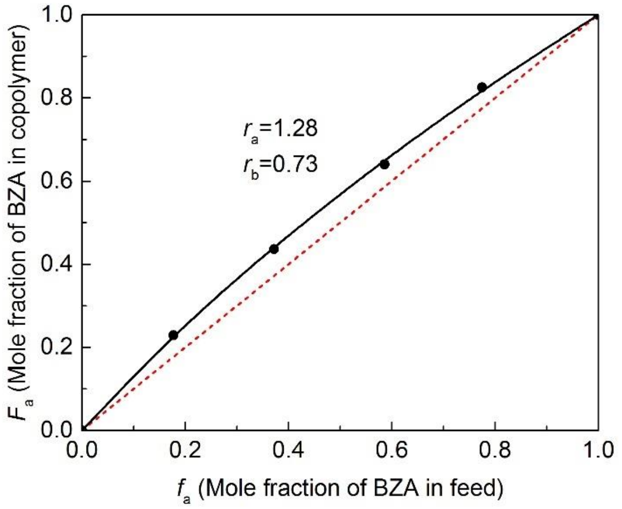

Figure S2. Plot of the mole fraction of BZA in copolymers versus that in the feed to determine the reactivity ratios of BZA/PEGA copolymers (**P1-P20**).

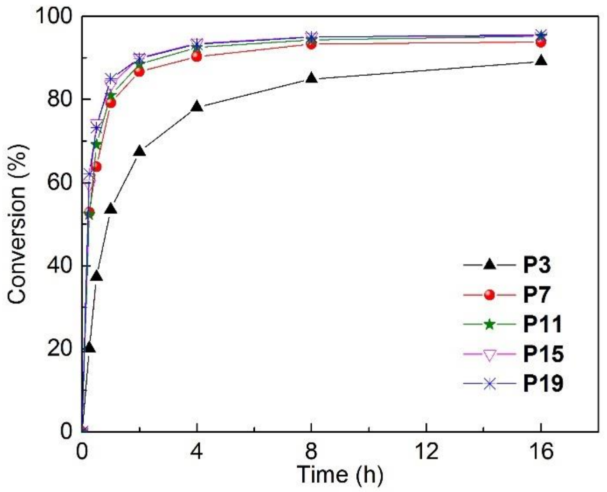

Figure S3. Plots of conversion versus time in the polymerization of BZA_m-$PEGA_n$ copolymers (**P3**, **P7**, **P11**, **P15**, and **P19**).

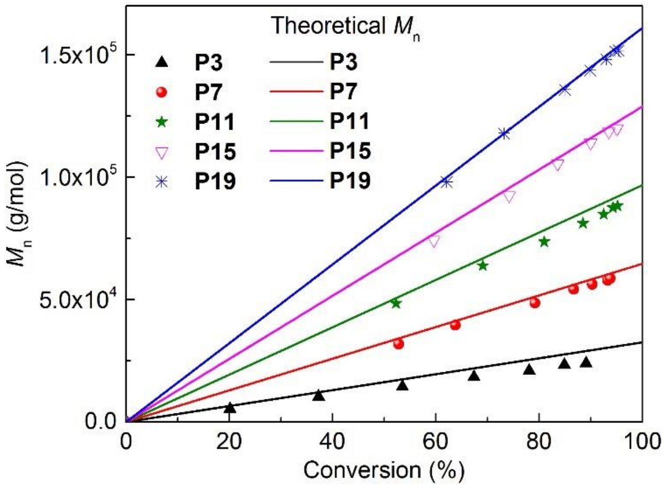

Figure S4. Evolution of molecular weight with conversion for BZA_m-$PEGA_n$ polymerizations (**P3**, **P7**, **P11**, **P15**, and **P19**). Solid lines indicate the theoretical number average molecular weight (M_n), assuming the formation on one living polymer per one radical initiator.

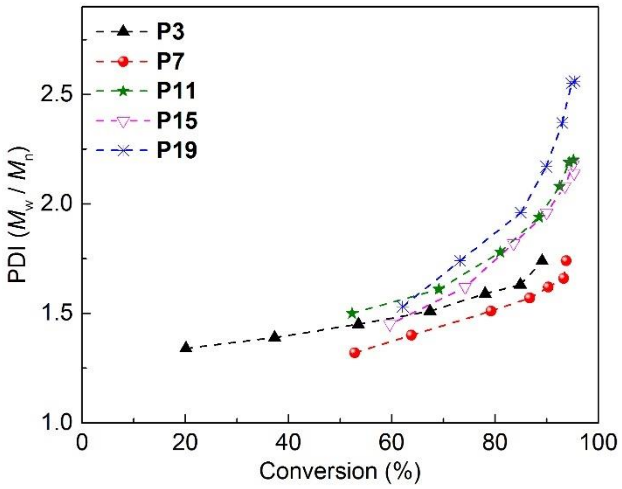

Figure S5. Evolution of polydispersity with conversion for BZA_m-$PEGA_n$ polymerizations (**P3**, **P7**, **P11**, **P15**, and **P19**).

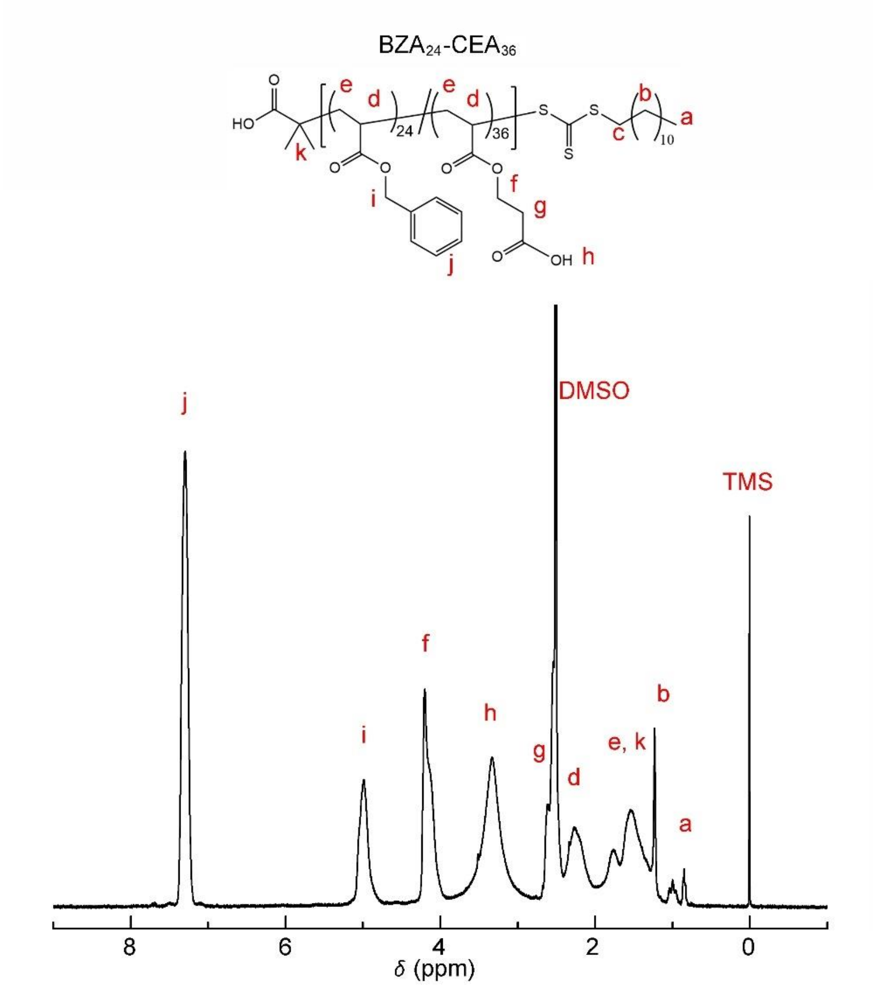

Figure S6. ^{1}H NMR spectrum of BZA_{24}-CEA_{36} in DMSO-d_6 at 25 °C.

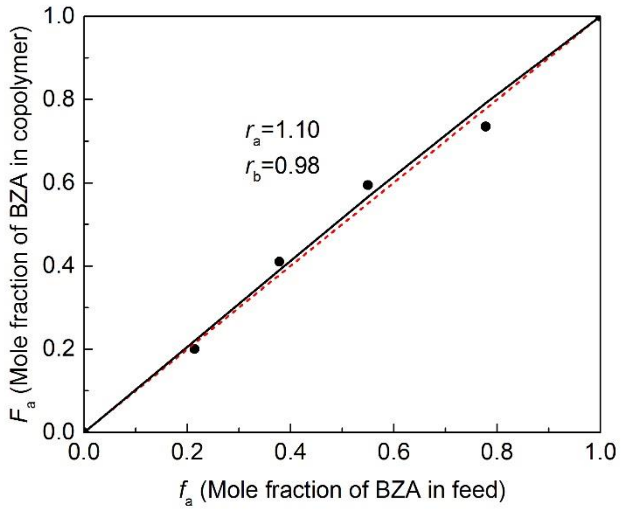

Figure S7. Plot of the mole fraction of BZA in copolymers versus that in the feed to determine the reactivity ratios of BZA/CEA copolymers.

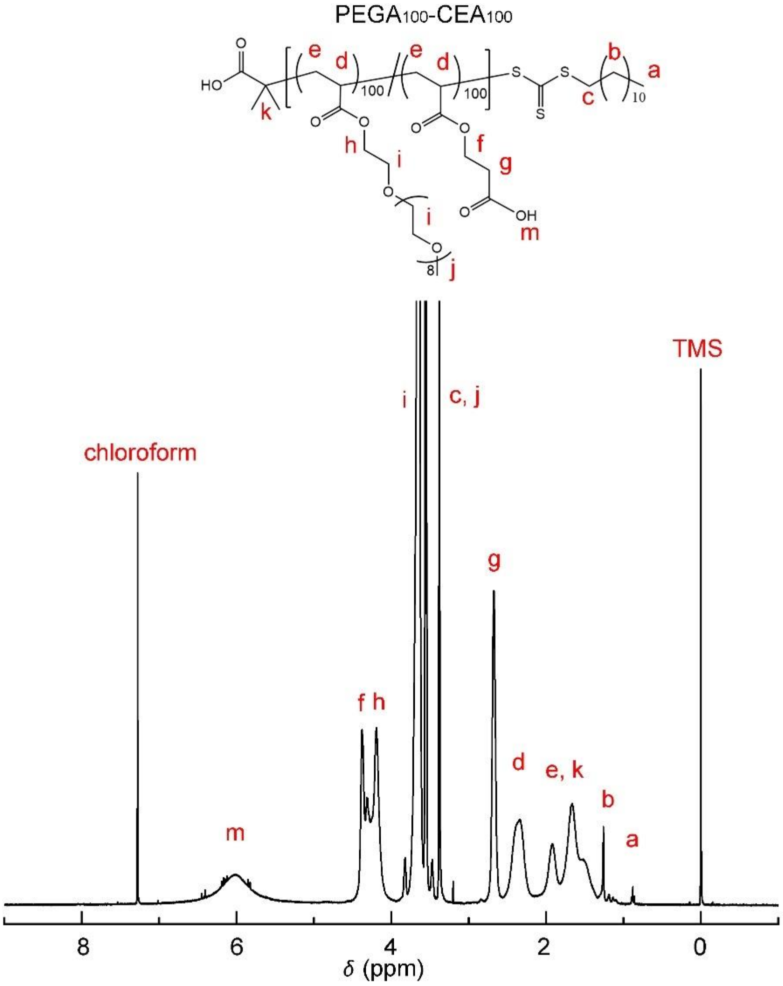

Figure S8. ^{1}H NMR spectrum of $PEGA_{100}$-CEA_{100} in chloroform-*d* at 25 °C.

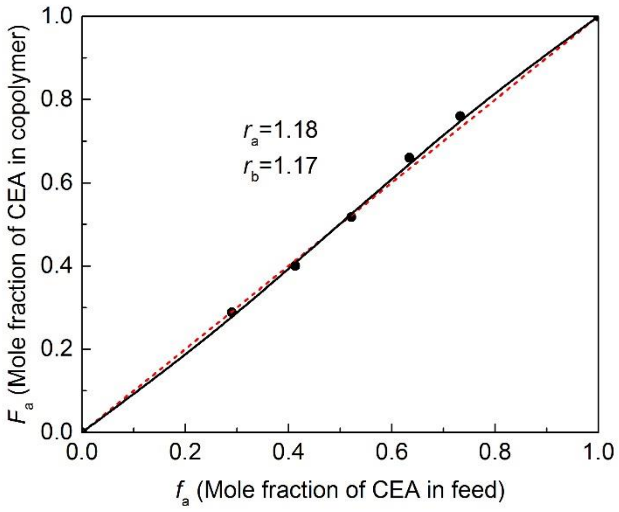

Figure S9. Plot of the mole fraction of CEA in copolymers versus that in the feed to determine the reactivity ratios of PEGA/CEA copolymers.

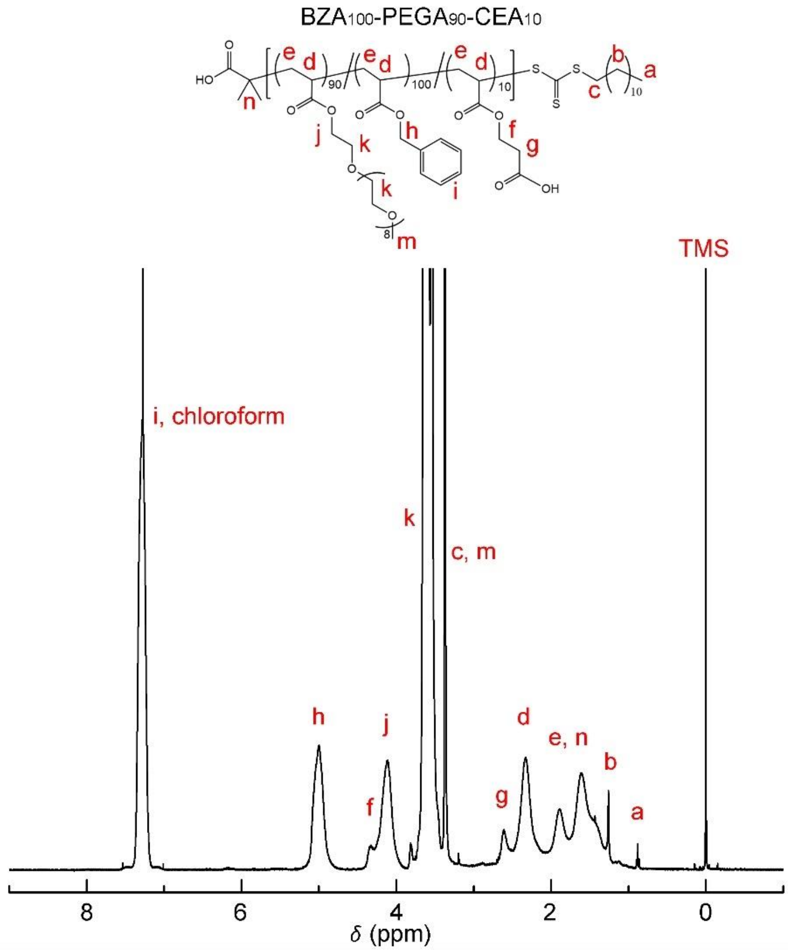

Figure S10. ^{1}H NMR spectrum of **TP1** in chloroform-*d* at 25 °C.

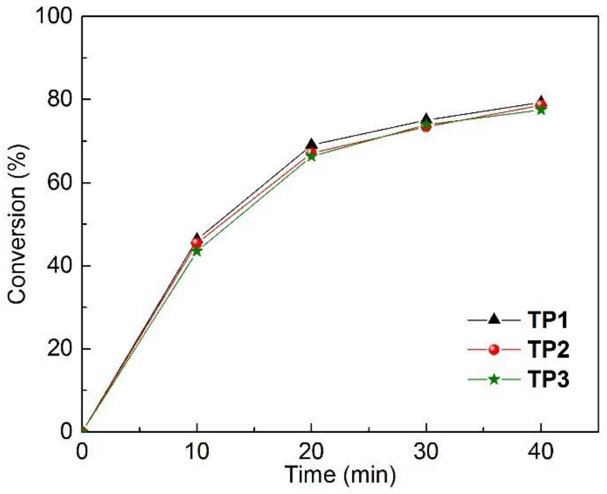

Figure S11. Plots of conversion versus time in the polymerization of **TP1-TP3**.

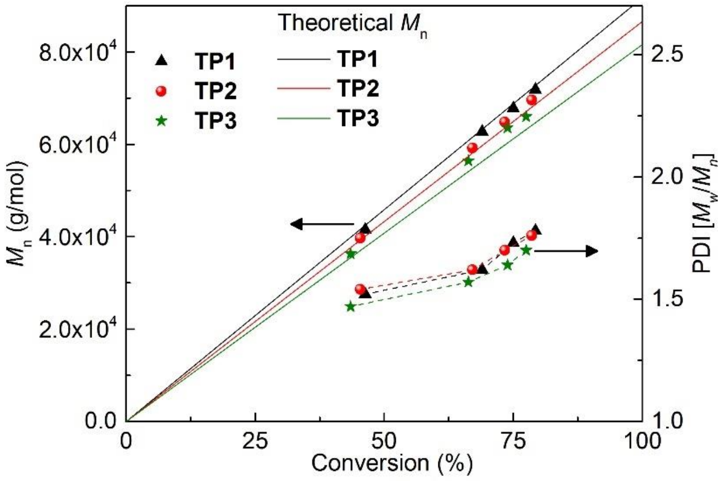

Figure S12. Evolution of molecular weight and polydispersity with conversion for **TP1-TP3** polymerizations. Solid lines indicate the theoretical number average molecular weight (M_n), assuming the formation on one living polymer per one radical initiator.

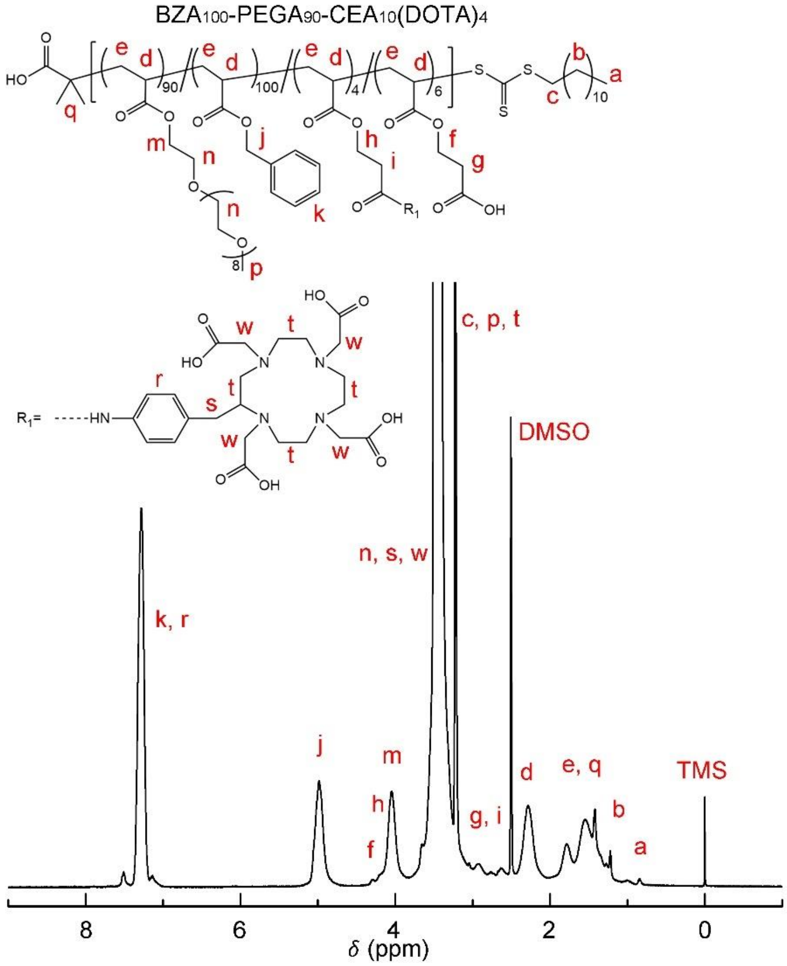

Figure S13. ^{1}H NMR spectrum of BZA_{100}-$PEGA_{90}$-$CEA_{10}(DOTA)_4$ in DMSO-d_6 at 25 °C.

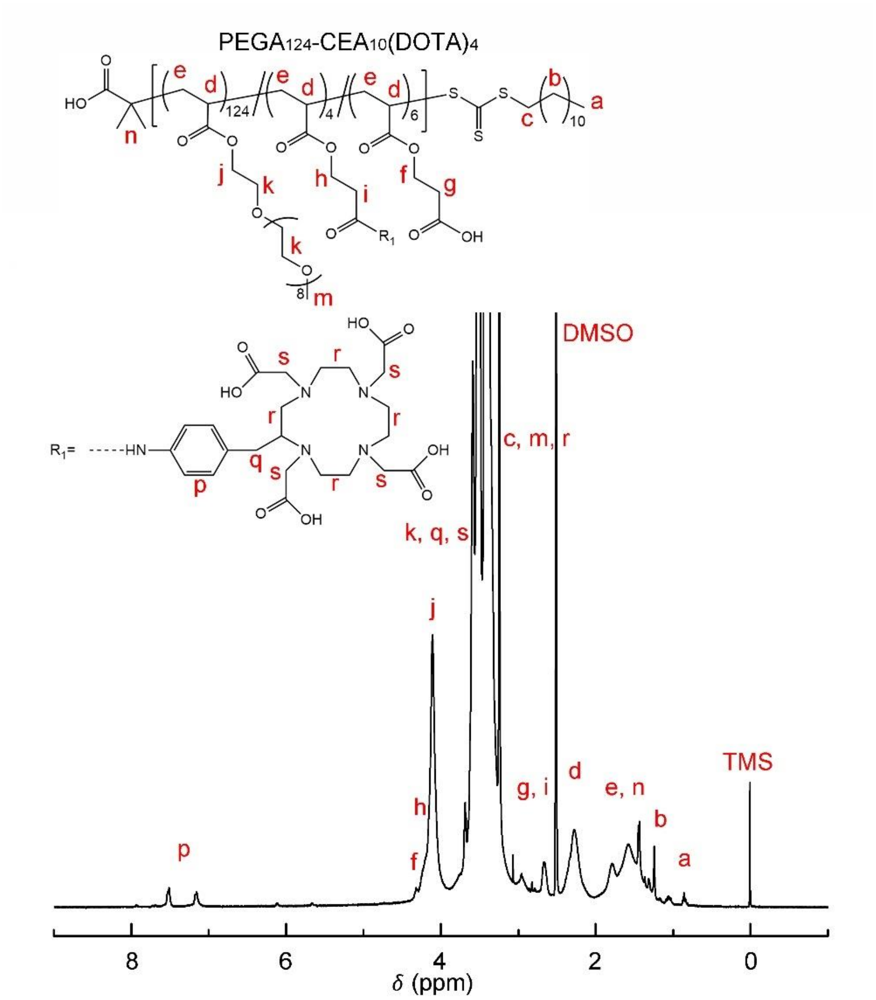

Figure S14. ^{1}H NMR spectrum of PEGA$_{124}$-CEA$_{10}$(DOTA)$_4$ in DMSO-d_6 at 25 °C.

.

5. Supporting data

5.1. Physical property of copolymers

Table S2. Characterization of random copolymers **P1-P20**.

No.	*dn*/*dc* in PBS[a)] [mL/g]	*dn*/*dc* in $CHCl_3$[b)] [mL/g]	M_w by MALS-PBS[c)] [g/mol]	M_w by MALS-$CHCl_3$[d)] [g/mol]	DA[e)]
**P1**	0.1311	0.0477	22,300	27,000	0.83
**P2**	0.1338	0.0603	26,000	17,100	1.52
**P3**	0.1396	0.0591	47,900	16,600	2.89
**P4**	0.1361	0.0698	149,500	17,600	8.49
**P5**	0.1357	0.0498	42,100	40,300	1.04
**P6**	0.1370	0.0609	47,600	42,100	1.13

**P7**	0.1336	0.0680	62,100	35,700	1.74
**P8**	0.1419	0.0697	104,500	37,900	2.76
**P9**	0.1300	0.0496	76,600	80,800	0.95
**P10**	0.1312	0.0596	74,400	71,300	1.04
**P11**	0.1297	0.0651	81,800	60,100	1.36
**P12**	0.1359	0.0684	110,000	64,400	1.71
**P13**	0.1192	0.0537	127,100	126,600	1.00
**P14**	0.1246	0.0519	118,200	130,200	0.91
**P15**	0.1354	0.0547	108,300	107,600	1.01
**P16**	0.1366	0.0686	139,000	97,800	1.42
**P17**	0.1210	0.0535	152,800	155,500	0.98
**P18**	0.1376	0.0641	145,800	149,000	0.98
**P19**	0.1365	0.0614	139,300	126,900	1.10
**P20**	0.1386	0.0701	144,700	140,700	1.03

[a)] Refractive index increment of copolymers in phosphate-buffered saline (PBS) solutions; [b)] Refractive index increment of copolymers in chloroform solutions; [c)] Absolute weight-average molecular weight measured by SEC-MALS in PBS; [d)] Absolute weight-average molecular weight measured by SEC-MALS in chloroform; [e)] Degree of aggregation (DA) in water: DA= M_w from SEC-MALS in PBS / M_w from SEC-MALS in chloroform.

.

Table S3. Characterization of random copolymers **TP4-TP6**.

No.	*dn/dc* in PBS[a)] [mL/g]	*dn/dc* in $CHCl_3$[b)] [mL/g]	M_w by MALS-PBS[c)] [g/mol]	M_w by MALS-$CHCl_3$[d)] [g/mol]	DA[e)]
**TP4**	0.1334	0.0691	82,100	89,900	0.91
**TP5**	0.1369	0.0718	75,700	86,000	0.88
**TP6**	0.1363	0.0778	74,900	142,400	0.53

[a)] Refractive index increment of copolymers in phosphate-buffered saline (PBS) solutions; [b)] Refractive index increment of copolymers in chloroform solutions; [c)] Absolute weight-average molecular weight measured by SEC-MALS in PBS; [d)] Absolute weight-average molecular weight measured by SEC-MALS in chloroform. [e)] Degree of aggregation (DA) in water: DA= M_w from SEC-MALS in PBS/M_w from SEC-MALS in chloroform.

5.2. Stability and Gd-leakage from SMDC-Gds.

PEGA-Gd4, SMDC-Gd4, and SMDC-Gd17 were dissolved in 5 mL of HEPES buffer (1 M, pH 7.4) containing 150 mM NaCl and 10 mg/mL BSA, and the initial concentration of Gd was measured by inductively coupled plasma mass spectrometry (ICP-MS) [Shimadzu ICPMS-2030 (Shimadzu Corporation, Kyoto, Japan)]. Then, each sample solution was put into a dialysis bag (Spectra/Por dialysis membrane, MWCO = 3.5 kD) and incubated with the same buffer (45 mL) at 37 °C. After 1, 2, 3, 5, and 10 days, Samples were analyzed by ICP-MS and the amount of Gd in SMDC-Gd was measured.

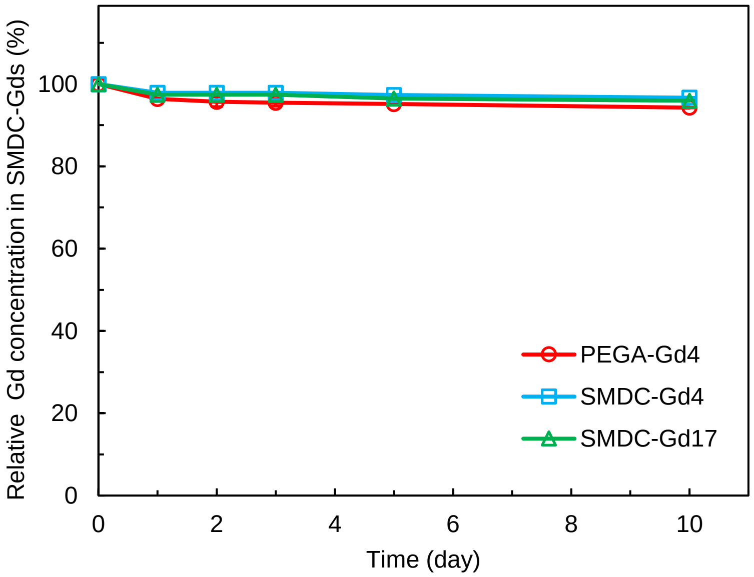

Figure S15. Gd-loaded polymers incubated in HEPES buffer (1 M, pH 7.4) with NaCl (150 mM) and BSA (10 mg/mL) at 37 °C maintained their incorporated Gd over 10 days. Data are means ± s.d., n =3.

5.2. Biodistribution of SMDC-Gds

The biodistribution of SMDC-Gds was investigated on CT26 tumor bearing BALB/c female mice (n=6), and the time profiles of Gd concentrations in the main organs (not illustrated in the main text) are shown in **Figures S15-S19**.

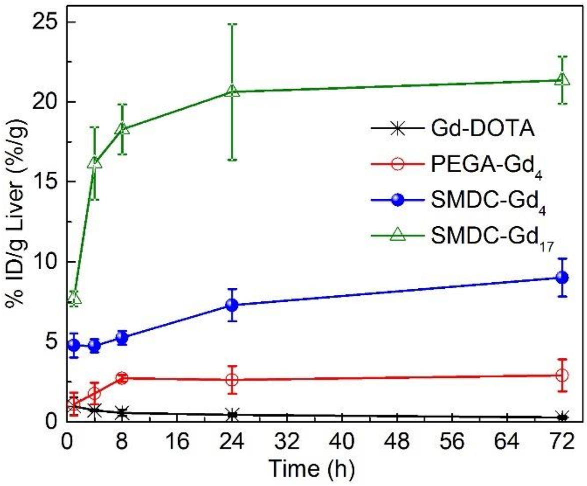

Figure S16. Time profiles of Gd concentration in the liver after intravenous injection.

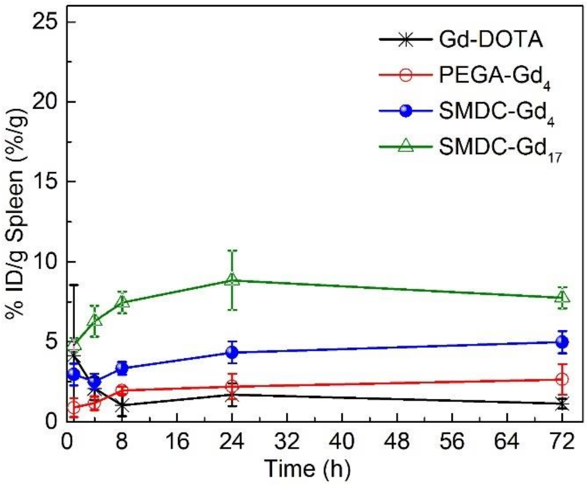

Figure S17. Time profiles of Gd concentration in the spleen after intravenous injection.

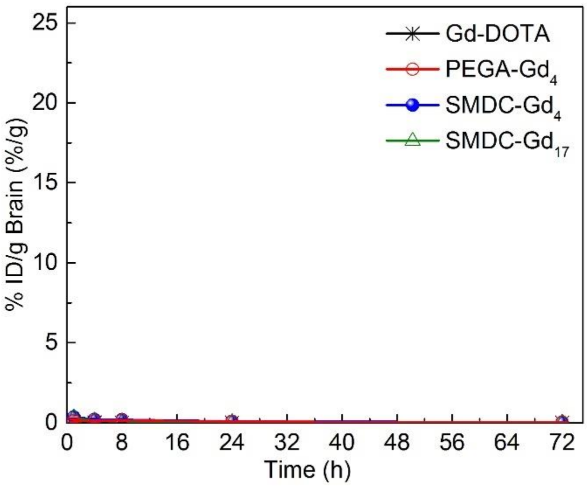

Figure S18. Time profiles of Gd concentration in the brain after intravenous injection.

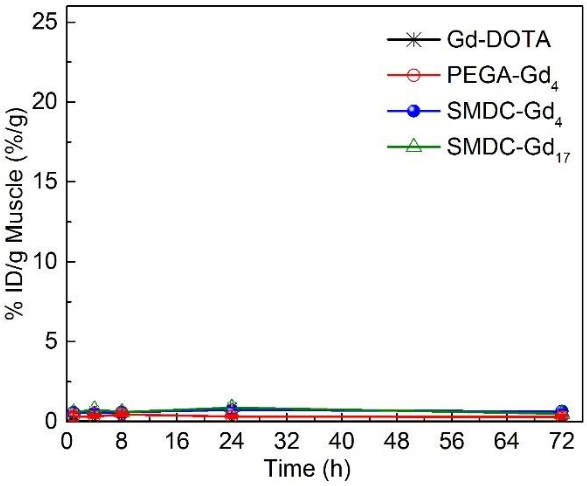

Figure S19. Time profiles of Gd concentration in the muscle after intravenous injection.

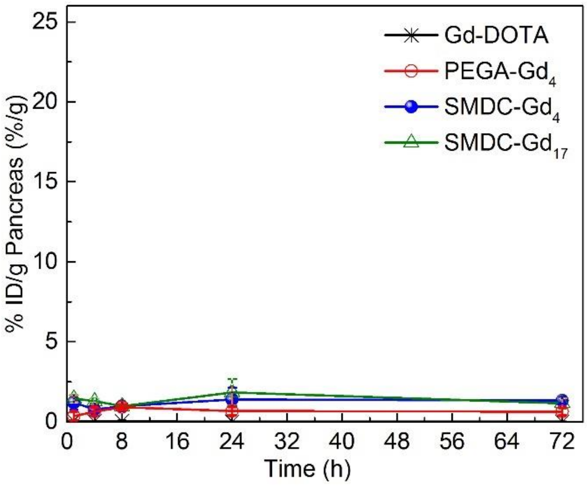

Figure S20. Time profiles of Gd concentration in the pancreas after intravenous injection.

5.3. Area under the curve ratios

The area under the curve (AUC) ratios of the tumor to main organs were calculated from the biodistribution result to clarify selectivity to the tumor.

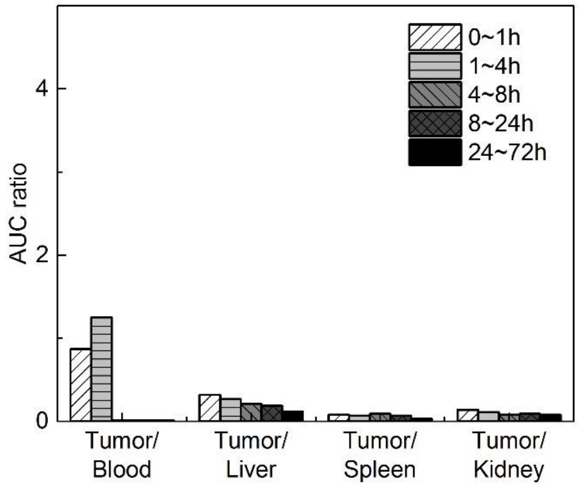

Figure S21. Area under the Gd concentration curve (AUC) ratios of the tumor to the main organs after the intravenous administration of Gd-DOTA.

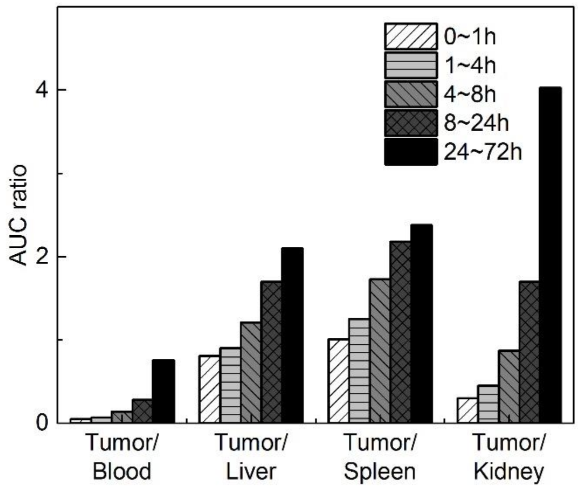

Figure S22. Area under the Gd concentration curve (AUC) ratios of the tumor to the main organs after the intravenous administration of PEGA-Gd_4.

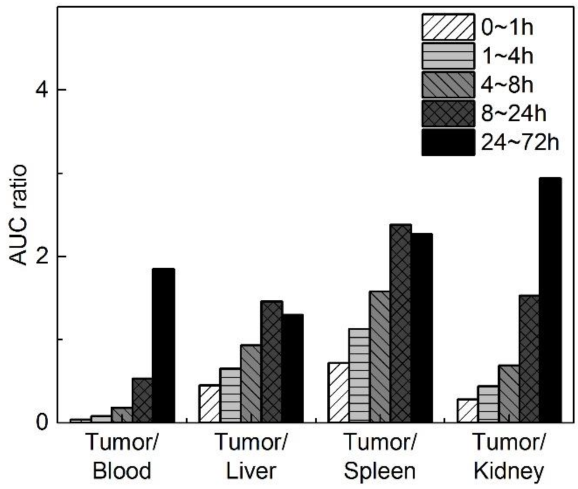

Figure S23. Area under the Gd concentration curve (AUC) ratios of the tumor to the main organs after the intravenous administration of SMDC-Gd_4.

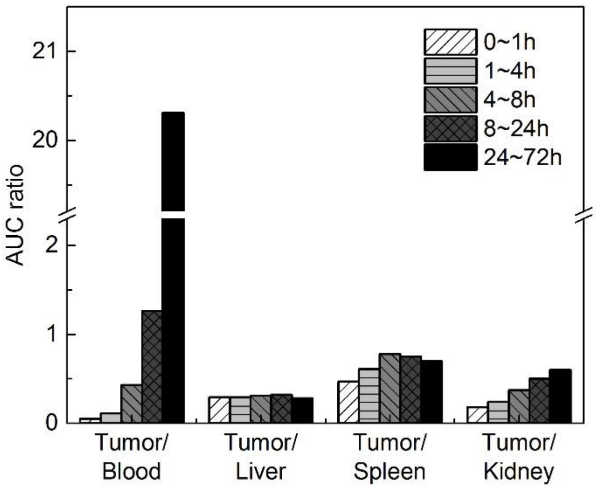

Figure S24. Area under the Gd concentration curve (AUC) ratios of the tumor to the main organs after the intravenous administration of SMDC-Gd_{17}.

5.4. Cytotoxicity assay

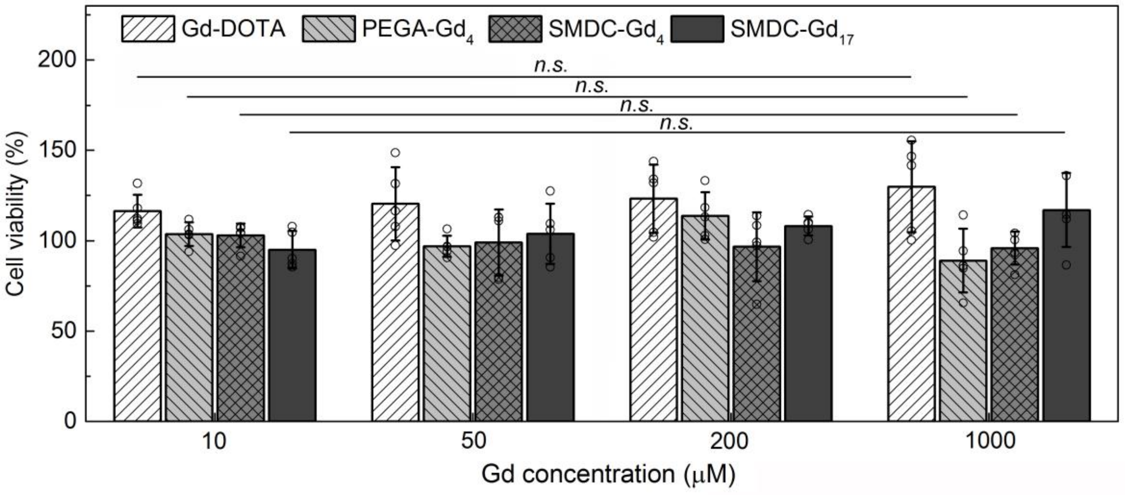

Figure S25. Cell viability of CT26 cells treated with Gd-conjugated contrast agents. *n.s.* $p \geq 0.05$.

5.5. Analysis of blood parameters

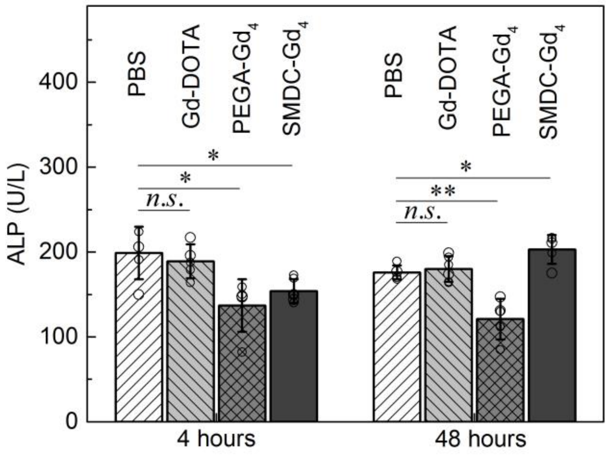

Figure S26. Effects of Gd-conjugated contrast agents on the alkaline phosphatase level in mice. *n.s.* $p \geq 0.05$, * $p < 0.05$, ** $p < 0.01$.

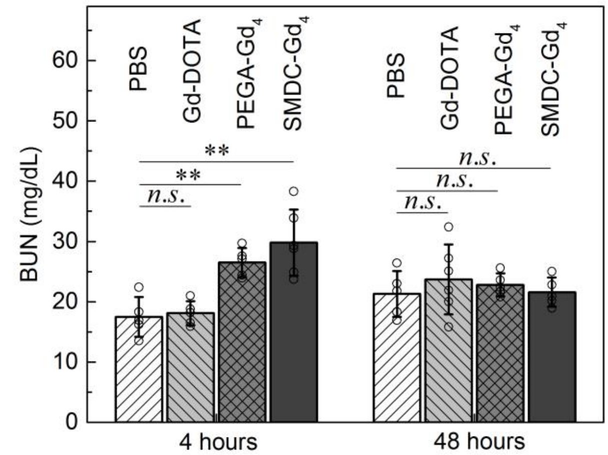

Figure S27. Effects of Gd-conjugated contrast agents on the blood urea nitrogen in mice. *n.s.* $p \geq 0.05$, ** $p < 0.01$.

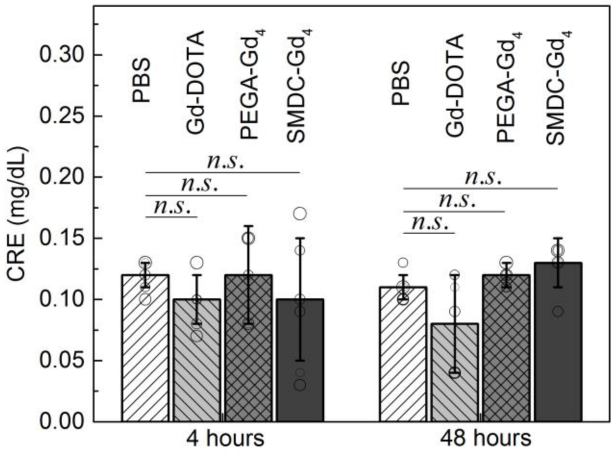

Figure S28. Effects of Gd-conjugated contrast agents on the creatinine level in mice. *n.s.* $p \geq 0.05$.

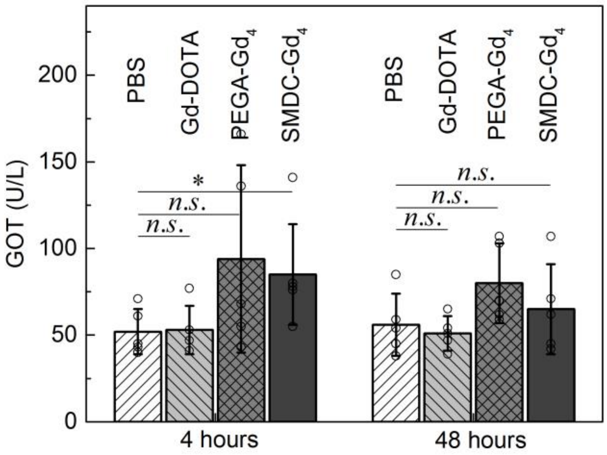

Figure S29. Effects of Gd-conjugated contrast agents on the glutamic-oxaloacetic transaminase level in mice. *n.s.* $p \geq 0.05$, * $p< 0.05$.

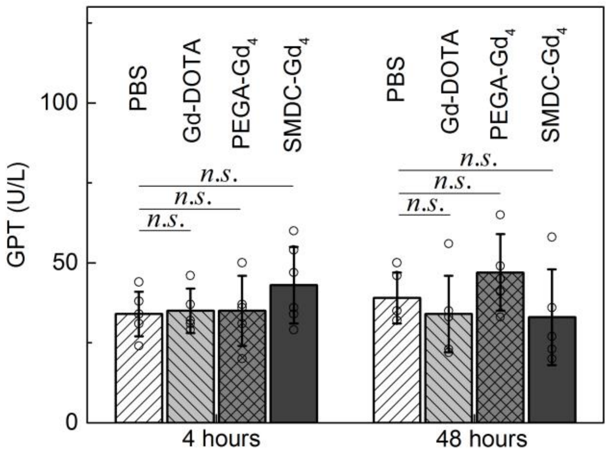

Figure S30. Effects of Gd-conjugated contrast agents on the glutamic-pyruvic transaminase level in mice. *n.s.* $p \geq 0.05$.

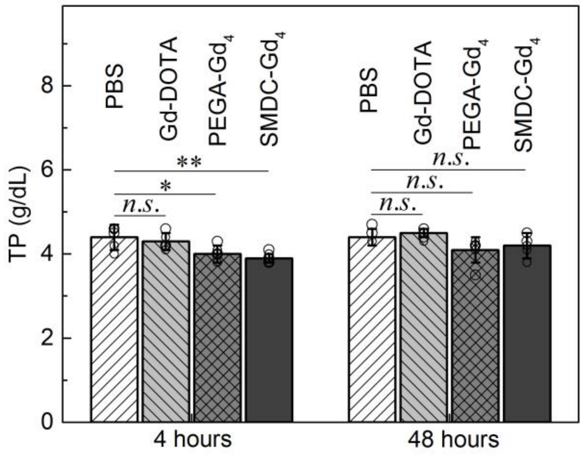

Figure S31. Effects of Gd-conjugated contrast agents on the total protein level in mice. *n.s.* $p \geq 0.05$, * $p< 0.05$, ** $p < 0.01$.

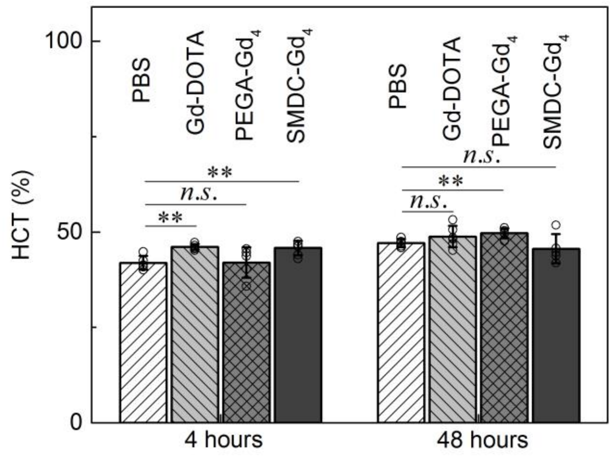

Figure S32. Effects of Gd-conjugated contrast agents on the hematocrit level in mice. *n.s.* $p \geq 0.05$, ** $p < 0.01$.

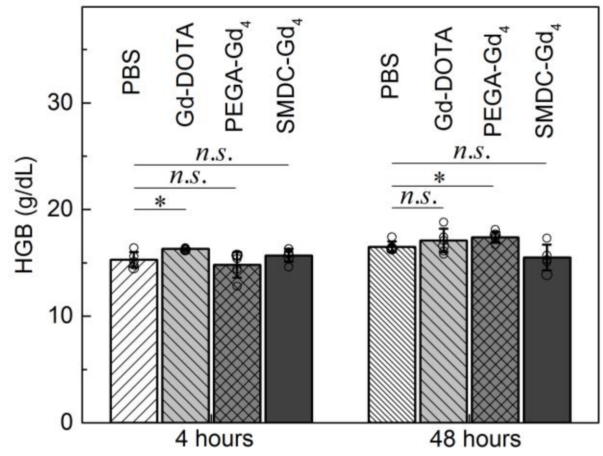

Figure S33. Effects of Gd-conjugated contrast agents on the hemoglobin level in mice. *n.s.* $p \geq 0.05$, * $p < 0.05$.

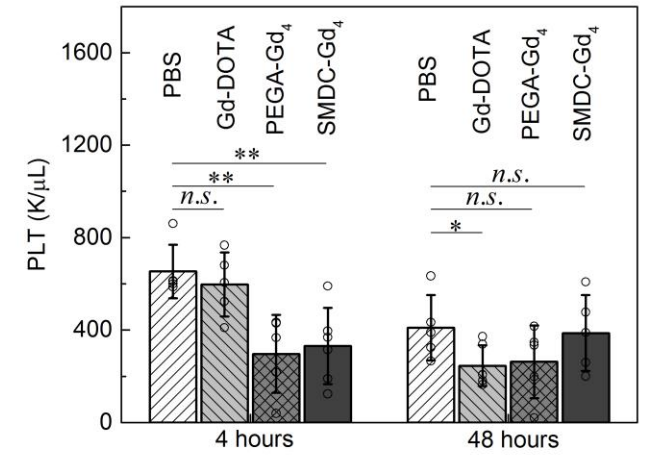

Figure S34. Effects of Gd-conjugated contrast agents on the platelet count in mice. *n.s.* $p \geq 0.05$, * $p< 0.05$, ** $p < 0.01$.

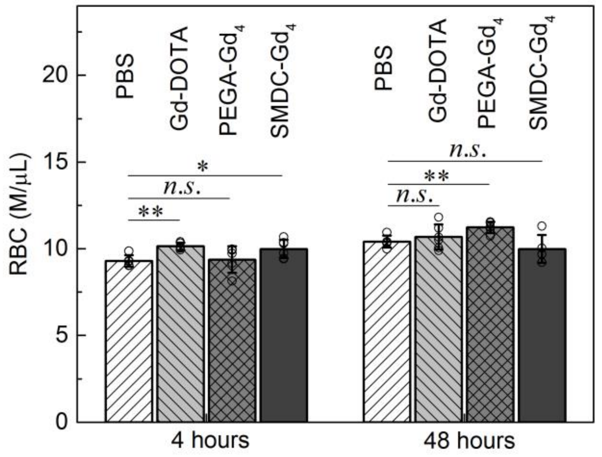

Figure S35. Effects of Gd-conjugated contrast agents on the red blood cell count in mice. *n.s.* $p \geq 0.05$, * $p< 0.05$, ** $p < 0.01$.

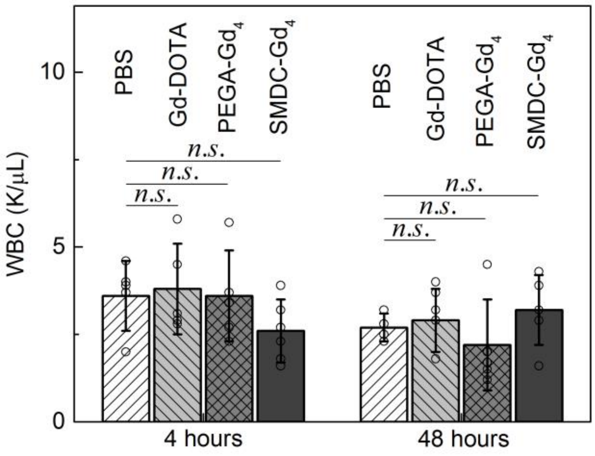

Figure S36. Effects of Gd-conjugated contrast agents on the white blood cell count in mice. *n.s.* $p \geq 0.05$.

.

5.6. Dose dependency of SMDC-Gds for magnetic resonance imaging (MRI)

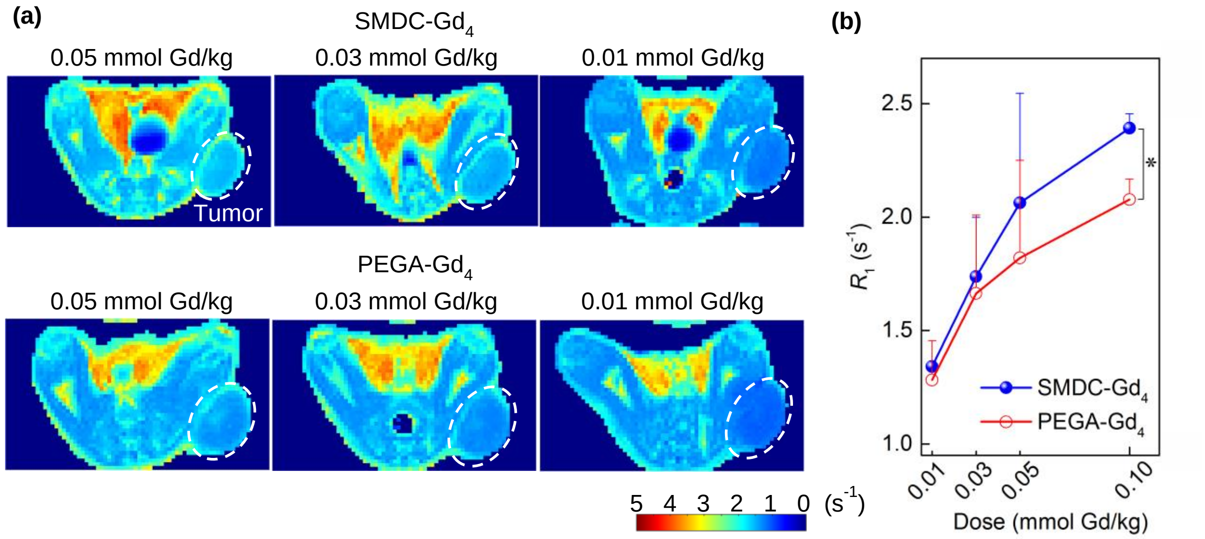

Figure S37. Contrast effect of SMDC-Gd_4 and PEGA-Gd_4 in MRI with multiple doses. (a) Longitudinal relaxation ratio (R_1) distribution maps of CT26 tumor bearing mice at T_1 MRI. (b) R_1 in tumor versus the dose curves of SMDC-Gd_4 and PEGA-Gd_4. Data are shown as mean ± SEM, n = 2-4, *$p < 0.05$. Comparison of R_1 values in tumor areas between SMDC-Gd_4 and PEGA-Gd_4. Adipose tissue showed the highest R_1 in all cases.

5.7. Effect of injection time on Gd concentration in organs

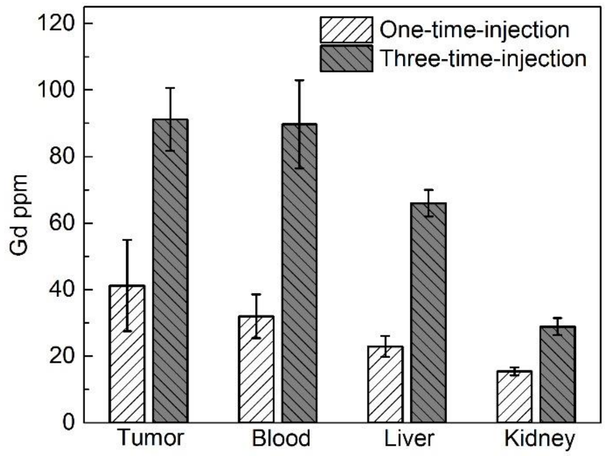

Figure S38. Gd concentrations in tumors and main organs after 24 h from last intravenous injection of SMDC-Gd_4.

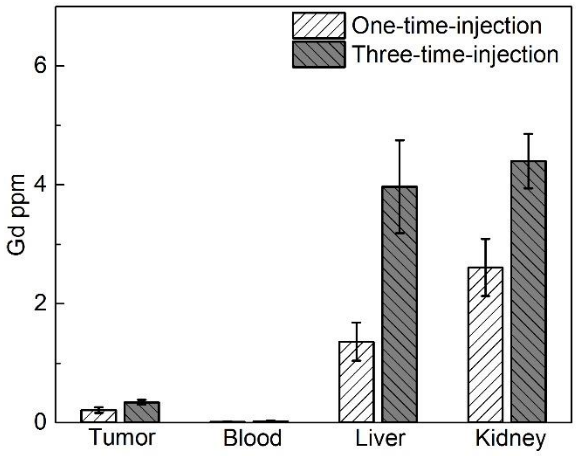

Figure S39. Gd concentrations in tumors and main organs after 24 h from last intravenous injection of Gd-DOTA.
